# A mouse model of Zhu-Tokita-Takenouchi-Kim syndrome reveals indispensable SON functions in organ development and hematopoiesis

**DOI:** 10.1172/jci.insight.175053

**Published:** 2024-01-30

**Authors:** Lana Vukadin, Bohye Park, Mostafa Mohamed, Huashi Li, Amr Elkholy, Alex Torrelli-Diljohn, Jung-Hyun Kim, Kyuho Jeong, James M. Murphy, Caitlin A. Harvey, Sophia Dunlap, Leah Gehrs, Hanna Lee, Hyung-Gyoon Kim, Jay Prakash Sah, Seth N. Lee, Denise Stanford, Robert A. Barrington, Jeremy B. Foote, Anna G. Sorace, Robert S. Welner, Blake E. Hildreth, Ssang-Taek Steve Lim, Eun-Young Erin Ahn

**Affiliations:** 1 Department of Pathology, Division of Molecular and Cellular Pathology, and; 2 Department of Neurosurgery, University of Alabama at Birmingham, Birmingham, Alabama, USA.; 3 Metastasis Branch, Division of Cancer Biology, National Cancer Center, Goyang, Gyeonggi-do, South Korea.; 4 Department of Medicine, College of Medicine, Dongguk University, Gyeongju, South Korea.; 5 Department of Radiology and; 6 Department of Medicine, Cystic Fibrosis Research Center, University of Alabama at Birmingham, Birmingham, Alabama, USA.; 7 Department of Microbiology and Immunology, College of Medicine, University of South Alabama, Mobile, Alabama, USA.; 8 Department of Microbiology,; 9 O’Neal Comprehensive Cancer Center, and; 10 Department of Medicine, Division of Hematology and Oncology, University of Alabama at Birmingham, Birmingham, Alabama, USA.

**Keywords:** Development, Genetics, Genetic diseases, Molecular genetics, Mouse models

## Abstract

Rare diseases are underrepresented in biomedical research, leading to insufficient awareness. Zhu-Tokita-Takenouchi-Kim (ZTTK) syndrome is a rare disease caused by genetic alterations that result in heterozygous loss of function of SON. While patients with ZTTK syndrome live with numerous symptoms, the lack of model organisms hampers our understanding of SON and this complex syndrome. Here, we developed *Son* haploinsufficiency (*Son^+/–^*) mice as a model of ZTTK syndrome and identified the indispensable roles of *Son* in organ development and hematopoiesis. *Son^+/–^* mice recapitulated clinical symptoms of ZTTK syndrome, including growth retardation, cognitive impairment, skeletal abnormalities, and kidney agenesis. Furthermore, we identified hematopoietic abnormalities in *Son^+/–^* mice, including leukopenia and immunoglobulin deficiency, similar to those observed in human patients. Surface marker analyses and single-cell transcriptome profiling of hematopoietic stem and progenitor cells revealed that *Son* haploinsufficiency shifted cell fate more toward the myeloid lineage but compromised lymphoid lineage development by reducing genes required for lymphoid and B cell lineage specification. Additionally, *Son* haploinsufficiency caused inappropriate activation of erythroid genes and impaired erythropoiesis. These findings highlight the importance of the full gene expression of *Son* in multiple organs. Our model serves as an invaluable research tool for this rare disease and related disorders associated with SON dysfunction.

## Abstract 2

SON haploinsufficient mice serve as an animal model of Zhu-Tokita-Takenouchi-Kim syndrome and identify indispensable SON functions in multi-organ development and hematopoiesis.

## Graphical abstract

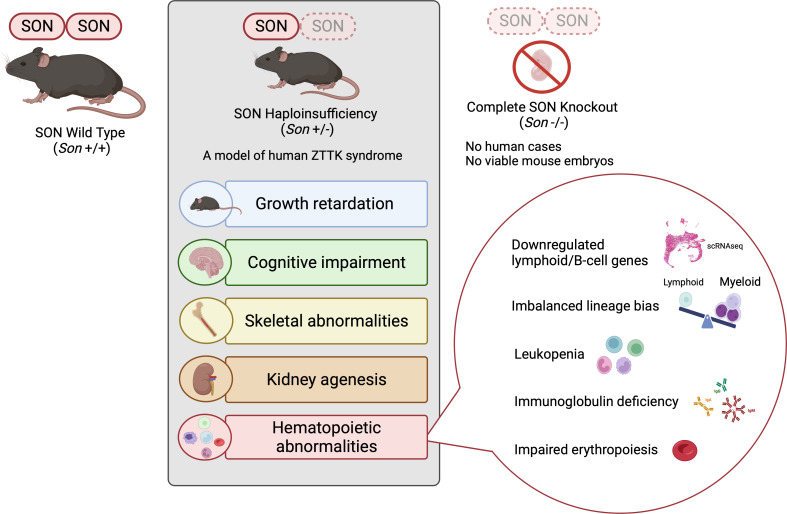

## Introduction

Rare diseases are clinical conditions with a low prevalence, affecting fewer than 200,000 people in the United States. However, there are more than 7,000 different types of rare diseases, and over 350 million individuals suffer from rare diseases globally, with more than half of them being children (1–3). Nevertheless, most rare diseases are set aside in a “dark zone” where attention from the medical and research community is lacking. Although advancements in whole-exome and -genome sequencing are rapidly unveiling previously undiagnosed rare genetic diseases, further research following the initial identification of these diseases is not sufficient to move forward (1, 4). This is partially due to a lack of proper model organisms that faithfully recapitulate the clinical features of human patients (5). Establishing proper animal models for newly identified human genetic diseases is critical to provide patients, families, and clinicians with further clinical characterization and to develop potential therapies. This effort is particularly critical to defining clinical features of rare diseases, which are often ambiguous because of the limited patient number.

Zhu-Tokita-Takenouchi-Kim (ZTTK) syndrome (also known as SON-related disorder) is a recently identified rare genetic disease characterized by developmental delay and multiple congenital anomalies (6–9). This syndrome is caused by mutations or entire/partial deletion of the *SON* gene from 1 allele, resulting in heterozygous loss of function (LoF) of *SON* (6, 7). After initial reports of 28 individuals with *SON* LoF variants in 2016, the number of cases continues to increase, revealing various clinical features that were not noticed previously. Although the initial effort focused on patients’ symptoms associated with developmental delay (DD) and intellectual disability (ID), further examination of the patients and subsequent research identified that *SON* LoF causes a wide spectrum of clinical features besides DD and ID (10–16). Thus, ZTTK syndrome is a complex, multisystem developmental disorder (OMIM 617140 and MedGen UID 934663).

Our initial discovery of this syndrome confirming the pathogenicity of *SON* LoF led to family connections, and our continuous support for the families greatly contributed to the launch of an official nonprofit organization, the ZTTK SON-Shine Foundation (https://zttksonshinefoundation.org/).

The *SON* gene is located on human chromosome 21 and encodes the SON protein, which possesses both DNA- and RNA-binding abilities and mainly localizes in nuclear speckles. Compromised SON function leads to aberrant and alternative RNA splicing, particularly for transcripts bearing weak splice sites (17–20), and SON-mediated RNA splicing is critical for maintaining pluripotency in embryonic stem cells (21). We have also shown that SON binds to DNA and suppresses H3K4me3 modifications at transcription start sites by interacting with Menin and sequestering it from the MLL1/2 methyltransferase complex (22). Recent attention to the role of nuclear speckles in gene expression has revealed that SON serves as the core of nuclear speckles (23), enhances p53-mediated transcription (24), and regulates 12-hour ultradian rhythm-associated proteostasis (25). Therefore, SON governs the expression of myriad target genes by regulating transcription, RNA splicing, and nuclear speckle assembly, while exerting its effects on specific sets of genes.

To extend our understanding of the clinical features associated with ZTTK syndrome and to study how *Son* loss affects developmental processes and growth, we created mouse models with the floxed *Son* gene, as well as mice with a germline *Son* deletion. Here, we report on the indispensable roles of *Son* in embryo development and demonstrate that mice with heterozygous *Son* loss recapitulate multiple clinical features of human ZTTK syndrome. Importantly, our mouse model identified hematopoietic abnormalities, which were found in patients with ZTTK syndrome. Moreover, we conducted surface marker–based phenotypic analysis combined with single-cell transcriptome analysis to determine how *Son* haploinsufficiency affects hematopoietic lineage determination and differentiation by perturbing critical gene expression.

## Results

### Generation of Son-floxed mice and constitutive Son-knockout mice.

**Figure 1:**
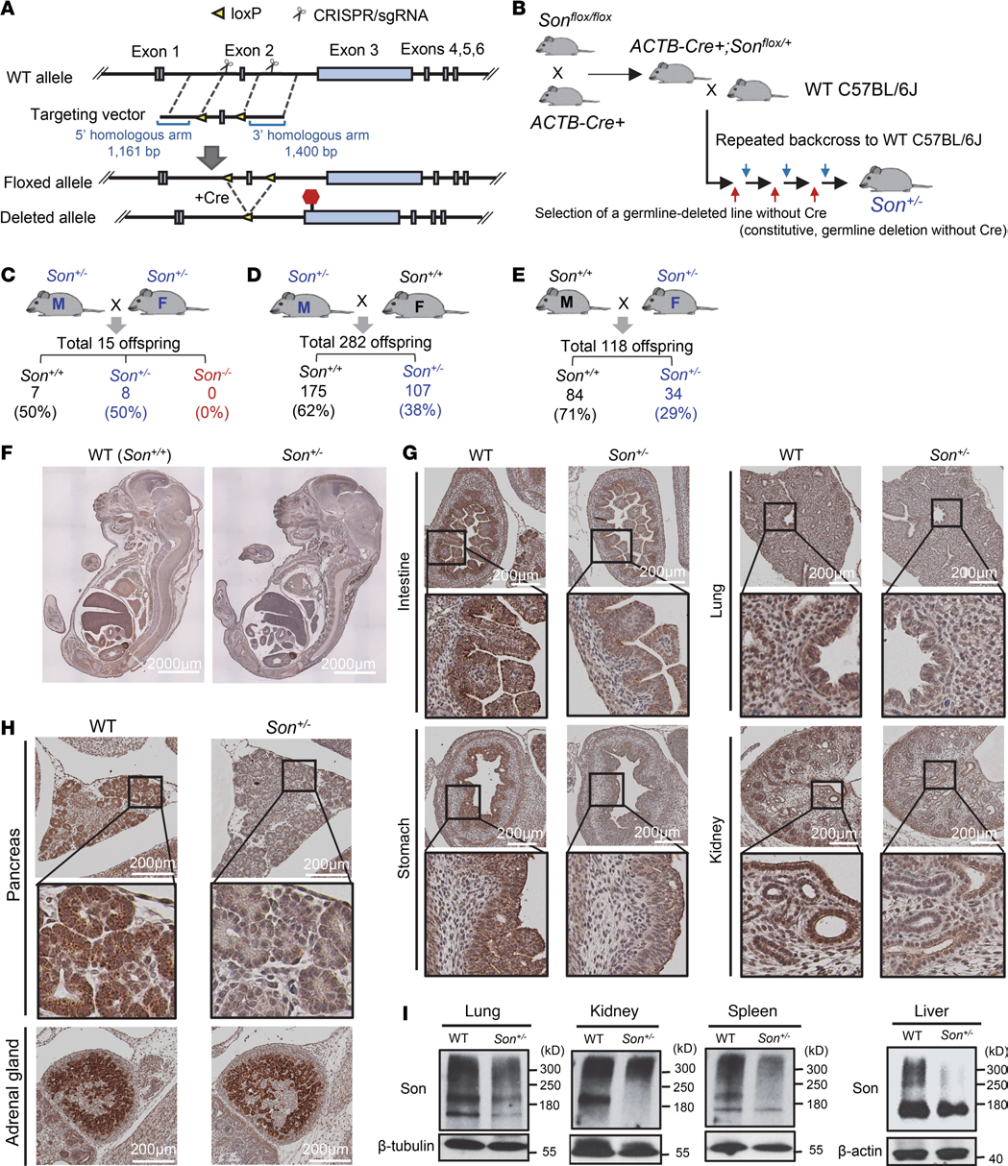
Generation of the *Son^+/−^* mouse models and characterization of Son expression patterns in embryos and organs. (**A**) Schematic diagram of the targeted locus and the strategy to insert *loxP* sites flanking *Son* exon 2. (**B**) A schematic illustrating the strategy to generate *Son^+/–^* mouse with permanent germline deletion of the floxed sequence. (**C**) *Son^+/–^* intercrossing revealed extremely low fertility of this breeding scheme and embryonic lethality of *Son^–/–^*. (**D**) Breeding strategy crossing male *Son^+/–^* with female WT mice, showing fewer *Son^+/–^* offspring than expected by the Mendelian ratio. (**E**) Breeding strategy crossing male WT with female *Son^+/–^* mice, showing further decreased *Son^+/–^* offspring. (**F**) IHC staining of Son in sagittal sections of WT and *Son^+/–^* embryos (E16). (**G** and **H**) IHC staining of Son in E16 embryonic organs with enlarged images of the boxed region. Original magnification, 20×. (**I**) Expression of the Son protein in WT and *Son^+/–^* mice was analyzed by Western blot. Loading control was β-tubulin or β-actin. Western blot data are representative of *n* = 3 independent experiments.

Given the absence of any reports on human cases with homozygous *Son* LoF variants, along with potential viability and fertility issues caused by heterozygous LoF of *Son*, we adopted a *Son*-floxed mouse model approach rather than a conventional knockout strategy. We inserted 2 *loxP* sites flanking the mouse *Son* exon 2 to generate *Son^fl/fl^* mice (Figure 1A and Supplemental Figure 1, A–C; supplemental material available online with this article; https://doi.org/10.1172/jci.insight.175053DS1). To create a mouse model that mimics human ZTTK syndrome, we proceeded to generate mice with heterozygous *Son* deletion in the entire body by crossing *Son^fl/fl^* mice with β-actin–cre (*ACTB-cre*) mice. After verifying that the *ACTB-Cre^+^* *Son^fl/wt^* mice were viable, we backcrossed them with wild-type (WT) C57BL/6J mice multiple times (>6 generations) to select the mice with germline deletion of the floxed sequence in the *Son* gene without Cre recombinase expression, which established the line with constitutive, heterozygous deletion of *Son* (*Son^+/–^*) from germline (Figure 1B and Supplemental Figure 1, D and E). Deletion of the floxed region resulted in a frameshift due to the loss of exon 2 and a subsequent premature termination codon in exon 3.

### Homozygous deletion of Son results in embryonic lethality while mice with heterozygous deletion of Son as a model for ZTTK syndrome were born and viable but not at a normal Mendelian ratio.

We performed a cross between a *Son^+/–^* male and a *Son^+/–^* female. However, this *Son^+/–^* intercrossing did not yield any *Son^–/–^* offspring (Figure 1C), indicating that constitutive, homozygous *Son* deletion (*Son* knockout; KO) causes embryonic lethality. To identify the embryonic stage at which lethality occurs, we collected numerous embryos at different stages of development. Despite our attempts to genotype as early as E6.5–7.5 (gastrulation), we did not detect any embryos showing the *Son^–/–^* genotype. These results indicate that *Son* is indispensable for mouse embryo development, and *Son*-null embryos with constitutive homozygous *Son* deletion lead to lethality earlier than the gastrulation stage. This aligns with the absence of identified patients with ZTTK syndrome with homozygous *SON* loss-of-function mutations. Additionally, we observed that *Son^+/–^* intercrossing breeding cages exhibited extremely low levels of fertility, yielding only 15 pups from over 10 breeding pairs attempted in 2 years (Figure 1C).

The *Son^+/–^* mice, modeling ZTTK syndrome, were born and viable but did not follow the expected Mendelian ratio. In the *Son^+/–^* intercrossing, the ratio of *Son^+/+^* and *Son^+/–^* offspring was around 1:1, which is not the expected Mendelian ratio 1:2 (Figure 1C). When *Son^+/–^* mice were crossed with *Son^+/+^* WT mice, the frequency of offspring was substantially lower than the expected 50% (Figure 1, D and E). Notably, the sex of the *Son^+/–^* breeder influenced the frequency of *Son^+/–^* offspring. When a *Son^+/–^* male was crossed with a WT female, about 38% of the offspring were *Son^+/–^* (Figure 1D), while *Son^+/–^* females crossed with WT males resulted in only 29% *Son^+/–^* offspring (Figure 1E). These findings suggest that constitutive, heterozygous *Son* deletion during embryonic development partially induces prenatal lethality, possibly exacerbated when the deleted allele is passed on maternally. It is also possible that the compromised production or viability of oocytes contributes to the difficulty of getting offspring from *Son^+/–^* female mice. Further studies are needed to determine the exact cause and molecular basis of these findings.

### Son is ubiquitously expressed, with the highest levels in the epithelial lining of gastrointestinal tracts and the gland structures in developing embryos, and a general reduction in Son expression was verified in Son^+/–^ embryos.

Next, we performed H&E staining of sagittal sections of whole embryos (E16–E17) to investigate the gross morphology of *Son^+/–^* embryos. We found no noticeable defects in the morphology of the *Son^+/–^* embryos compared with *Son^+/+^* (Supplemental Figure 2A). Immunohistochemistry (IHC) staining for Son verified that *Son^+/–^* embryos had reduced Son protein levels throughout the body compared with *Son^+/+^* WT embryos (Figure 1F). We found that while Son protein was ubiquitously expressed in the embryo, certain tissues/organs showed particularly high levels of expression. These included gastrointestinal epithelial cells such as intestinal epithelium crypts and villi (with higher levels in the crypts) and inner layers of the stomach, the lung (particularly in airway epithelial cells), and the kidney (tubular epithelial cells) (Figure 1G and Supplemental Figure 2B). We also found that organs with gland structures, such as pancreatic islets, thyroid glands, and adrenal glands, exhibited high levels of Son protein expression (Figure 1H and Supplemental Figure 2B). Relatively moderate to low levels of Son were detected in cartilage, circular muscle layers of gastrointestinal tracts, and the ventricular zone of the brain (Figure 1F).

Interestingly, the majority of tissues/organs with high Son expression levels were endoderm-derived epithelial linings and glands, while mesoderm- and ectoderm-derived tissues/organs showed lower levels of Son expression. The negative control without primary antibodies was completely devoid of brown staining, demonstrating the specificity of our staining (Supplemental Figure 2C). Our extensive IHC analyses verified the reduction of Son in *Son^+/–^* embryos and provided valuable information on the endogenous Son protein expression pattern in developing mouse embryos.

### Examining Son^+/–^ mice reveals various patterns and sizes of the Son protein expressed among different tissues/organs.

Previous studies, both from our group and others, have shown that multiple bands are detected in Son Western blot experiments, ranging from over 300 kDa to approximately 120 kDa, in several human cell lines as well as human peripheral blood mononuclear cells (6, 17, 26, 27). To examine the Son protein expression, we performed Western blotting on different tissues/organs isolated from *Son^+/–^* mice and compared the patterns with those from *Son^+/+^* WT mice (Figure 1I and Supplemental Figure 2D). While we detected bands around and above the 300 kDa molecular weight marker, which should represent the full-length Son protein, we also observed a band migrating to a position slightly below the 180 kDa marker. Interestingly, we detected different band patterns in different tissues/organs, and all bands showed decreased intensity in tissues/organs from *Son^+/–^* mice (Figure 1I). Therefore, it is likely that all of these bands represent specific forms of the Son protein that are decreased in our *Son^+/–^* mouse model.

### Son^+/–^ mice show marked growth retardation and fail to gain weight properly, which resembles the symptoms found in patients with ZTTK syndrome.

**Figure 2:**
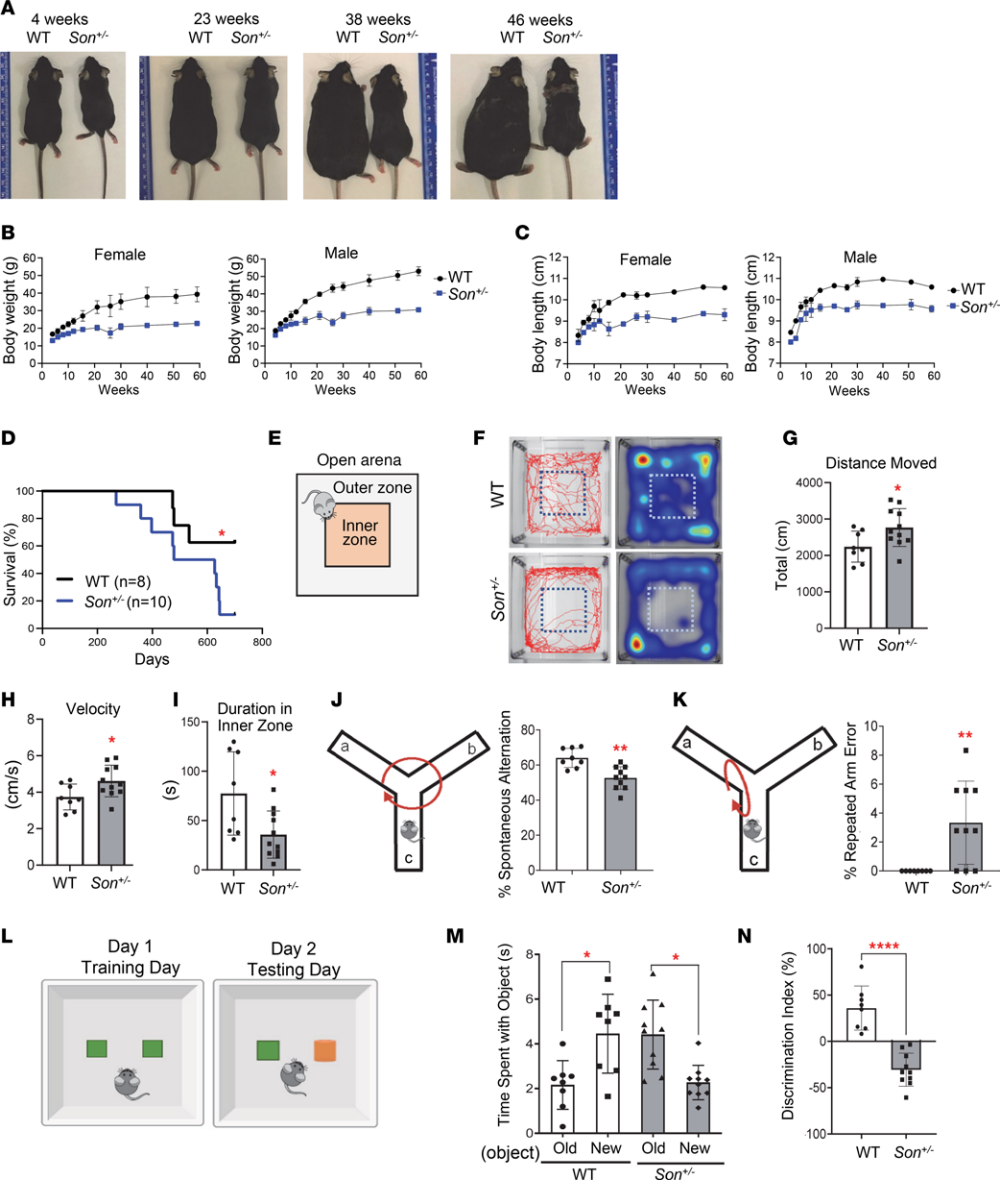
*Son^+/–^* mice show growth retardation, weight gain failure, and behavioral phenotypes, resembling clinical features of human ZTTK syndrome. (**A**) Representative images indicating body size differences between WT and *Son^+/–^* mice at various ages. (**B** and **C**) The body weight (**B**) and the body length (**C**) of female and male mice were measured at different ages up to 60 weeks. Data are presented as mean ± SD, *n* = 5–12. (**D**) Kaplan-Meier curves of survival data in *Son^+/–^* mice compared with WT with a follow-up duration of 700 days of age; *P < 0.05 by log-rank test. (**E**) Schematic depicting the open field test. (**F**) Representative tracks of WT and *Son^+/–^* showing the total distance traveled by the indicated mouse and the representative heatmaps of time the mouse spent in the open arena. (**G**) The total distance moved was calculated by measuring the total centimeters traveled. (**H**) Velocity was calculated by measuring centimeters traveled per second (cm/s). (**I**) Time (seconds) spent in the inner zone of the open area. (**J** and **K**) Schematics and the graphs of percentage level showing correct spontaneous alternation (**J**) and incorrect spontaneous alternation, also called repeated arm error (**K**), of WT and *Son^+/–^* mice in the Y-maze test. (**L**) Schematic depicting the experimental design of the novel object recognition test. Old object, depicted in green; new object, depicted in orange. (**M**) Time spent with the old versus new object was calculated in seconds. (**N**) A discrimination index was calculated using the percentage of the ratio between the time spent (T) per exploration number (N) of the novel object by the time spent per exploration number of both objects (Tnew/Nnew – Told/Nold/Tnew/Nnew + Told/Nold). For graphs in **G**–**K**, **M**, and **N**, data are presented as mean ± SD, *n* = 8–10 per group. *P ≤ 0.05, **P < 0.01, ****P < 0.0001 by 2-tailed *t* test.

Since growth retardation is a common phenotype observed in humans with ZTTK syndrome (11), we monitored the growth of *Son^+/–^* mice and their WT (*Son^+/+^*) littermates and found growth retardation of *Son^+/–^* mice, which persisted and worsened throughout their lifetime. Both male and female *Son^+/–^* mice gradually gained weight but not as much as their WT littermates. Notably, after 21 weeks of age, *Son^+/–^* mice barely gained body weight, with the average weight gained during the age of 21 to 60 weeks being only 3.3 g for males and 2.5 g for females. Meanwhile, male and female WT littermates gained 13.2 g and 7.3 g, respectively, during the same period. At 60 weeks of age, the body weights of male and female *Son^+/–^* mice were approximately only 58% of those of male and female WT littermates (Figure 2, A and B, and Supplemental Figure 3 for measurement data).

We also measured the body length of *Son^+/–^* mice and their WT littermates between the ages of 4 and 60 weeks. During this time, the body length of *Son^+/–^* mice and WT littermates was about 90%–94% of WT body length from ages 4–12 weeks and remained 86%–90% of WT body length between ages 12 and 60 weeks (Figure 2C and Supplemental Figure 3 for measurement data). These analyses demonstrated that *Son^+/–^* mice showed growth retardation, particularly a failure to gain weight, recapitulating the phenotype of patients with ZTTK syndrome.

Despite growth retardation and failure to gain weight, *Son^+/–^* mice did not show noticeable mortality until 300–400 days. However, after this time point, they showed an increased death compared with their WT littermates when monitored for up to 700 days, under standard care in the pathogen-free animal facility (Figure 2D). The cause of the increased death at this point was not identifiable through gross examination of organs and tissues.

### Son^+/–^ mice show signs of hyperactivity, anxiety-like behavior, and cognitive impairment.

To investigate whether *Son^+/–^* mice show any phenotypic traits associated with cognitive and behavioral abnormalities, which are prominent in adults with ZTTK syndrome (11), we conducted tests on a cohort of 8- to 10-month-old mice. We first performed the open field test (Figure 2E), which measures overall locomotor activity, exploratory habits, and anxiety-like behavior (28, 29). We tracked mouse activity across an “open field” arena with a center inner zone (Figure 2F). Compared with WT mice, *Son^+/–^* mice covered a significantly greater distance and had higher velocity (Figure 2, G and H), indicating hyperactivity or anxiety-related behaviors. We also measured thigmotaxis, which is the tendency to remain close to walls and is associated with anxiety-related behaviors (30). Interestingly, *Son^+/–^* mice spent more time close to the wall and less time in the open field compared with WT mice (Figure 2, F and I), suggesting that *Son^+/–^* mice have anxiety-like characteristics.

Next, we evaluated whether *Son^+/–^* mice exhibited signs of cognitive impairment using the Y-maze and novel object recognition tasks. In the Y-maze test, mice should be able to memorize the previously visited arm and prefer to explore new arms (a phenomenon known as “spontaneous alternation”) (31). We found that *Son^+/–^* mice exhibited a significant decrease in the percentage of spontaneous alternations (Figure 2J) and an increased frequency of repeatedly visiting the same arm (repeated arm errors) (Figure 2K), indicating deficits in spatial short-term working memory. To further assess their learning ability and memory, we employed the novel object recognition test (Figure 2L) (32). Interestingly, while WT mice spent more time with the novel object than the previously explored object, *Son^+/–^* mice did not show this innate preference for a novel object and spent less time exploring the novel object and more time with the familiar object (Figure 2, M and N), indicating an impairment in preferential recognition of the novel object.

### Son^+/–^ mice recapitulate many other clinical features of human ZTTK syndrome, including scoliosis/kyphosis, kidney hypoplasia/agenesis, and mild microcephaly.

**Figure 3:**
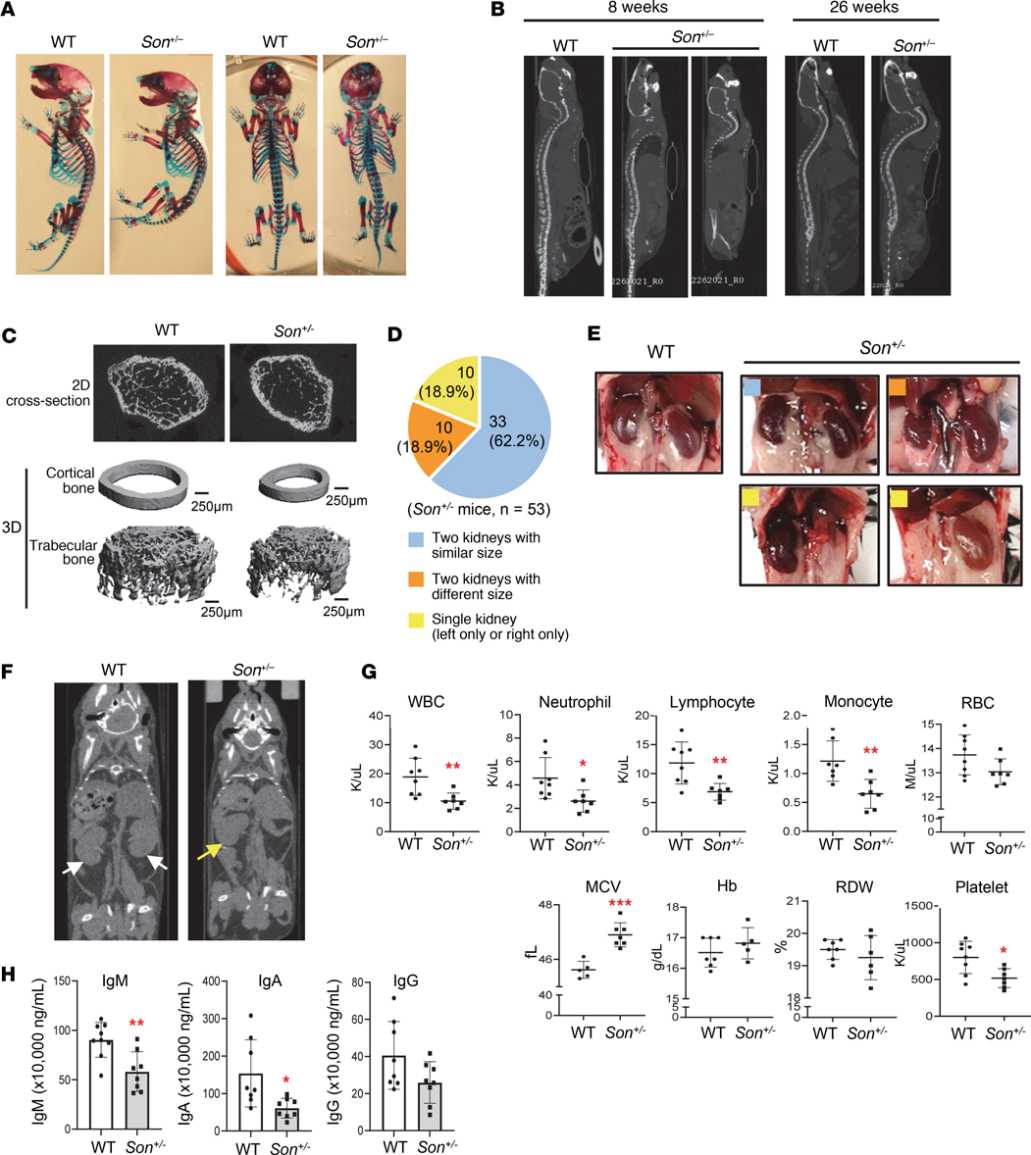
*Son^+/–^* mice faithfully recapitulate clinical features observed in human ZTTK syndrome, including scoliosis/kyphosis, kidney hypoplasia/agenesis, and hematological abnormalities. (**A**) Alizarin red and Alcian blue staining of WT and *Son^+/–^* mouse skeletons (postnatal day 1) showing signs of scoliosis and kyphosis. (**B**) Sagittal view of the μCT images showing kyphosis in *Son^+/–^* mice, which worsened as they aged. (**C**) 2D section and 3D reconstruction for the μCT scan of the femurs of 6-week-old WT and *Son^+/–^* mice. (**D**) Pie chart indicating the percentage of the indicated kidney status determined in total 53 *Son^+/–^* mice. (**E**) Representative images of normal kidneys from WT mice, and normal and abnormal kidneys observed in *Son^+/–^* mice. Color-coded squares shown (**D**) were marked on *Son^+/–^* kidney images to indicate the kidney types. (**F**) Coronal view of the μCT images of WT and *Son^+/–^* mice, indicating the presence of 2 kidneys in a WT mouse (white arrows) and 1 kidney in a *Son^+/–^* mouse (a yellow arrow). (**G**) Complete blood counts (CBCs) from whole blood of WT and *Son^+/–^* mice at 13 weeks of age. (**H**) ELISA analysis of immunoglobulins (IgM, IgA, and IgG) in the plasma of WT and *Son^+/–^* mice. (**G** and **H**) Data are presented as mean ± SD, *n* = 7–8. *P < 0.05, **P < 0.01, ***P < 0.001 by 2-tailed *t* test.

Another common phenotype observed in ZTTK syndrome is spine curvature, such as scoliosis and kyphosis (6, 7, 11). Alizarin red/Alcian blue staining of skeletons from 1-day-old pups showed curved spines in *Son^+/–^* mice, which mimic scoliosis and kyphosis, while no significant abnormalities in ossification were found (Figure 3A). Micro-computed tomography (μCT) imaging of 8-week-old *Son^+/–^* mice showed spine curvature, which became more severe as they aged (26 weeks), particularly for the cervical and thoracic curves (Figure 3B).

To examine the bone structure, the distal femoral metaphysis was examined by 2D and 3D reconstruction from μCT images (Figure 3C). The results revealed a marked reduction in the trabecular bone volume fraction (BV/TV) in *Son^+/–^* mice when compared with control littermates (Supplemental Figure 4). A decrease in trabecular number (Tb.N) and an increase in trabecular spacing were also observed in *Son^+/–^* mice, particularly in the triangulation plate model of analysis (Supplemental Figure 4). Based on similar trabecular thickness between groups, this indicates that reduced Tb.N resulted in reduced BV/TV in *Son^+/–^* mice. During cortical bone analysis in the midshaft of the femur of *Son^+/–^* mice, the μCT images also showed that the cross-sectional area of the bone was smaller than that of WT mice (Figure 3C). These findings revealed that *Son^+/–^* mice had reduced bone.

To examine whether *Son^+/–^* mice recapitulate the kidney phenotype of ZTTK syndrome (10), we analyzed 53 *Son^+/–^* mice for their kidney features. Among them, 10 mice (18.9%) had a single kidney (left or right only), and 10 mice (18.9%) had 2 kidneys with 1 hypoplastic kidney (Figure 3, D–F). The H&E staining images showed minimal to mild tubular generation/necrosis and mild interstitial edema in the samples from mice with a single kidney (Supplemental Figure 5). Our previous analysis indicated that 37.38% of patients with ZTTK syndrome previously examined for their kidneys (17 out of 45 patients) showed abnormal renal morphology (10, 11). Interestingly, 37.73% of the *Son^+/–^* mice we examined (20 out of 53) had a single kidney or 1 hypoplastic kidney (Figure 3F), indicating a similar level of phenotype penetrance in humans and mice.

Furthermore, an MRI of the brain showed that *Son^+/–^* mice had decreased total brain volume compared with WT littermates (Supplemental Figure 6), which recapitulates the microcephaly phenotype in patients with ZTTK syndrome (11).

Taken together, these findings indicate that *Son^+/–^* mice are an animal model that can recapitulate the skeletal, renal, and brain phenotypes observed in humans with ZTTK syndrome and could be invaluable research tools to study these abnormalities.

### A wide spectrum of hematological abnormalities has been observed in children with ZTTK syndrome, and Son^+/–^ mice show similar abnormalities in peripheral blood analysis and immunoglobulin measurements.

The clinical features of ZTTK syndrome include immunoglobulin deficiency and frequent infections (6, 7, 11). We collected voluntarily reported clinical features from families to the ZTTK SON-Shine Foundation website, which include a wide range of other hematological abnormalities, including bone marrow failure, high mean corpuscular volume (MCV), polycythemia, severe anemia, and thrombosis. Some children had to receive a transfusion because of low platelet levels. Additionally, low WBC counts, especially low levels of lymphocytes, neutrophils, and monocytes, have been identified from the complete blood count (CBC) (https://zttksonshinefoundation.org/; summarized in Supplemental Table 1). Interestingly, CBC analyses of *Son^+/–^* mice identified significant decreases in WBC (leukopenia) and low levels of lymphocytes, neutrophils, and monocytes (Figure 3G). RBC counts, red cell distribution width, and hemoglobin levels were normal, but high MCV levels (macrocytosis) and low platelet counts in *Son^+/–^* mice were observed (Figure 3G and Supplemental Figure 7). We also observed significant decreases in immunoglobulins, especially IgM and IgA, in the plasma of *Son^+/–^* mice even in the steady state (Figure 3H). These findings indicate that *Son^+/–^* mice recapitulate the hematological/immunological features found in humans with ZTTK syndrome.

### Son^+/–^ fetal liver hematopoiesis shows phenotypic alterations of HSPCs with a decreased pool of early-stage HSPCs and an increased bias toward megakaryocyte/erythroid lineages.

**Figure 4:**
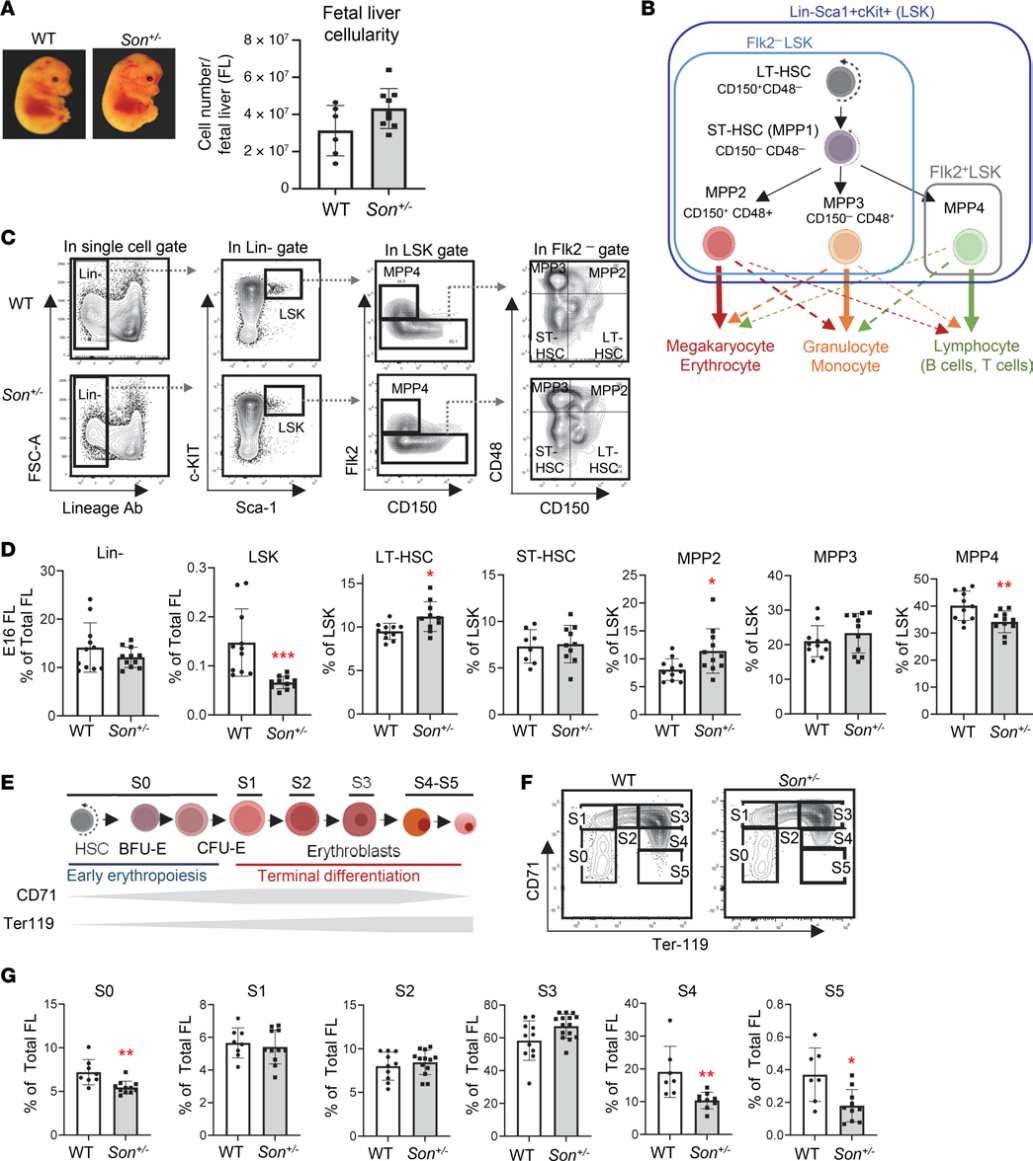
*Son* haploinsufficiency causes a reduction of the early-stage HSPC (LSK) pool size, an imbalance of myeloid versus lymphoid lineage–biased MPPs, and impaired erythroid terminal differentiation during fetal liver hematopoiesis in *Son^+/–^* embryos. (**A**) Representative image of WT and *Son^+/–^* embryos at E14 (left panel). Fetal liver cellularity at E14 (right panel) showing no statistically significant difference between WT and *Son^+/–^* fetal livers. (**B**) A schematic indicating subpopulations within the LSK population (LT-HSC, short-term HSC [ST-HSC], MPP2, MPP3, and MPP4), their lineage bias, and the surface markers. (**C**) Representative flow cytometry contour plots showing the gating scheme used to identify LSK and other subpopulations in the fetal liver. (**D**) Frequency of the indicated populations within total fetal liver (E16) cells (for Lin^–^ and LSK) or within the LSK population (LT-HSC, ST-HSC, MPP2, MPP3, and MPP4). (**E**) A schematic depicting the process of erythroid differentiation and the expression levels of CD71 and Ter119. (**F**) Representative flow cytometry contour plots demonstrating fetal liver erythroid subsets S0–S5 indicated by CD71 and Ter119 expression (E14). (**G**) Frequency of the indicated erythroid subsets, presented as percentage of total fetal liver (E14) cells. (**A**, **D**, and **G**) Data are presented as mean ± SD, *n* = 8–11. *P < 0.05, **P < 0.01, ***P < 0.001 by 2-tailed *t* test.

To verify that the hematopoietic features found in ZTTK syndrome are due to *SON* LoF, we further analyzed the hematopoiesis of our *Son^+/–^* mice. Since many hematological abnormalities can be attributed to altered phenotypes of early-stage hematopoietic stem and progenitor cells (HSPCs), we first analyzed HSPCs during fetal liver hematopoiesis. While fetal liver cellularity did not show significant changes (Figure 4A), we found that the percentage of early-stage HSPCs (defined by the absence of lineage markers and positivity of cKit and Sca1 expression; Lin*^–^*Sca1^+^cKit^+^ cells; denoted as LSK cells) was significantly decreased in the *Son^+/–^* fetal liver (Figure 4, B–D, and Supplemental Figure 8A).

Next, phenotypic hematopoietic stem cells (HSCs) and lineage-biased multipotent progenitors (MPPs) were analyzed from the *Son^+/–^* fetal liver using Flk2 and 2 signaling lymphocyte activation molecule family markers, CD48 and CD150 (33) (Figure 4, B and C). We observed a mild increase in long-term hematopoietic stem cells (Flk2*^–^*CD150^+^CD48*^–^* LSK cells; denoted as LT-HSCs) in the *Son^+/–^* fetal livers. More interestingly, among lineage-biased MPPs, megakaryocyte/erythroid (MegE) lineage–biased MPPs (Flk2^−^CD150^+^CD48^+^ LSK cells; denoted as MPP2) were increased, while lymphoid lineage–biased MPPs (Flk2^+^CD150^−^ LSK cells; denoted as MPP4) were decreased in both E14 and E16 *Son^+/–^* fetal livers (Figure 4, C and D, and Supplemental Figure 8A). These changes were marginal, but statistically significant, suggesting that HSPC subpopulations were already altered during fetal liver hematopoiesis.

We further analyzed the downstream myeloid progenitors (Lin*^–^*cKit^+^Sca1*^–^*; denoted as LK [Sca1^–^] cells) based on a classical model of myeloid progenitor differentiation (34) (Supplemental Figure 8B). We found that the LK (Sca1^–^) population was decreased, and the megakaryocyte/erythroid progenitor (MEP) population showed an increase (Supplemental Figure 8, C and D). Taken together, our data suggest that *Son* haploinsufficiency leads to an early-stage HSPC lineage disposition toward the MegE lineage during fetal liver hematopoiesis.

### Terminal erythroid differentiation is impaired during fetal liver hematopoiesis in Son^+/–^ embryos.

From the fetal liver, we next examined erythroid lineage cells in 6 different stages (S0–S5) based on CD71 (transferrin receptor) and Ter119 expression (Figure 4E). Our analysis revealed that erythroid progenitors with colony-forming potential (BFU-E and CFU-E) and erythroblasts up to the S3 stage showed similar levels of cell numbers in the WT and *Son^+/–^* fetal liver. However, we found a significant reduction in the S4 and S5 stages in the *Son^+/–^* fetal liver (Figure 4, F and G). These results indicate that early-stage erythroid progenitors are intact, and the onset of erythroid differentiation normally occurs in the *Son^+/–^* fetal liver. However, the late stage of terminal differentiation with downregulation of CD71 is impaired. Our analyses suggest that high MCV and anemia observed in ZTTK syndrome could be due to impaired erythroid terminal differentiation caused by *Son* haploinsufficiency.

### The bone marrow of Son^+/–^ mice showed a reduction in early-stage HSPC population and altered lineage bias with expansion of myeloid lineage–biased MPPs and reduction of lymphoid lineage–biased MPPs.

**Figure 5:**
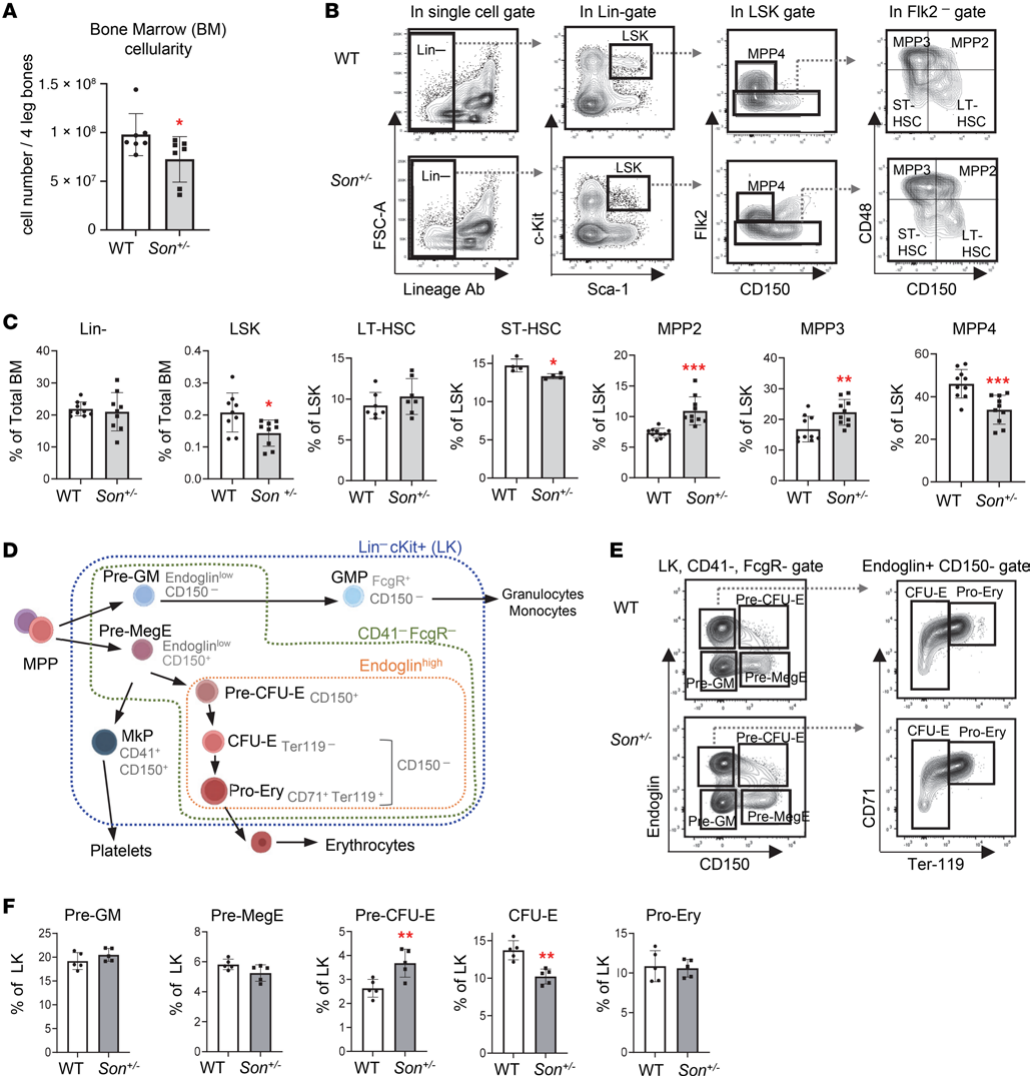
*Son* haploinsufficiency causes a reduction of the early-stage HSPC (LSK) pool size, increased myeloid lineage–biased MPPs, decreased lymphoid lineage–biased MPPs, and increased early-stage erythroid progenitors in the bone marrow of adult *Son^+/–^* mice. (**A**) Bone marrow cellularity from 8- to 10-week-old mice, presented as mean ± SD. *n* = 7, *P < 0.05 by 2-tailed *t* test. (**B**) Representative flow cytometry contour plots showing the gating scheme used to identify LSK and other subpopulations within the LSK pool of adult bone marrow. (**C**) Frequency of bone marrow LSK subsets presented as mean ± SD, *n* = 9; *P < 0.05, **P < 0.01, ***P < 0.001 by 2-tailed *t* test. (**D**) A schematic depicting differentiation of myeloerythroid progenitors (Lin^–^cKit^+^ [LK] cells) and surface markers defining the lineages and stages. (**E**) Flow cytometry contour plots demonstrating the gating scheme to define myeloerythroid progenitor subpopulations within CD41^–^FcgR^–^ LK cells, using Endoglin, CD150, CD71, and Ter119 markers. (**F**) Frequency of bone marrow myeloerythroid subsets within LK cells, presented as mean ± SD, *n* = 5; **P < 0.01 by 2-tailed *t* test.

Next, we analyzed the bone marrow of *Son^+/–^* mice (8–10 weeks), which showed an overall reduction of bone marrow cellularity compared with that of WT mice (Figure 5A). We found that the portion of LSK cells (early-stage HSPCs) was decreased in the bone marrow of *Son^+/–^* mice (Figure 5, B and C), which is similar to our observation from the fetal liver (Figure 4D). In addition, within the LSK population of *Son^+/–^* mice, we found an expansion of MegE-biased MPP2 and granulocyte/monocyte-biased (GM-biased) MPP3 and a reduction of lymphoid lineage–biased MPP4 (Figure 5C). These data, together with our fetal liver analyses, revealed that *Son* haploinsufficiency decreased the overall size of early-stage HSPCs (LSK cells), and there was a skewed bias in the MPP lineage toward the myeloid lineages (MegE and GM lineages) rather than the lymphoid lineage. Particularly, MPP2 expansion and MPP4 reduction were already initiated during fetal liver hematopoiesis and persisted in adult bone marrow hematopoiesis.

We further examined myeloid progenitor development, using CD150, FcgR, endoglin (CD105), CD41, CD71, and Ter119, and delineated functionally distinct myeloid-erythroid progenitor cells within LK cells (35) (Figure 5D). We did not see notable changes in granulocyte/monocyte precursors (pre-GM and GMP) and megakaryocyte progenitors (MkP). However, early-stage erythroid progenitors with colony-forming potential (pre–CFU-E) were increased whereas the next-stage progenitors, CFU-E, were decreased in the *Son^+/–^* bone marrow (Figure 5, E and F, and Supplemental Figure 9, A and B). Taken together, these analyses revealed that while *Son^+/–^* mice have an increase in MegE lineage–biased MPPs (MPP2), they have a compromised ability to move through the erythroid lineage differentiation process.

### Son^+/–^ mice show impaired B cell maturation in the spleen.

**Figure 6:**
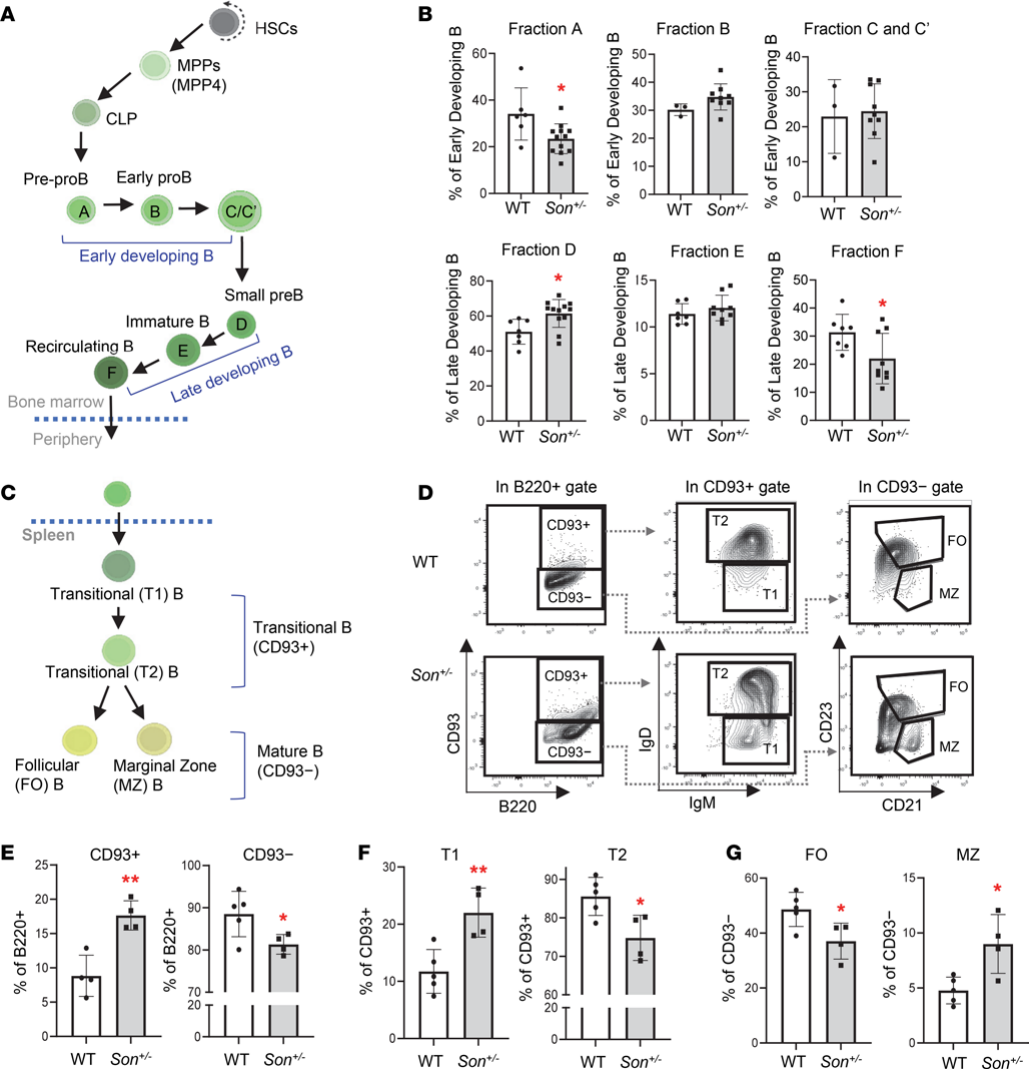
*Son* haploinsufficiency causes impaired B cell differentiation in the spleen of *Son^+/–^* mice. (**A**) A schematic depicting B cell development in the bone marrow. (**B**) Frequency of bone marrow B cell subsets (Hardy fractions) determined by flow cytometry analysis of surface markers. Data are expressed as mean ± SD, *n* = 3–12. *P < 0.05, by 2-tailed *t* test. (**C**) A schematic depicting B cell differentiation into transitional B and subsequently mature naive B cells in the spleen. (**D**) Flow cytometry contour plots demonstrating the gating scheme of CD93^+^ (transitional) and CD93^−^ (naive mature) B cells within the spleen B220^+^ cell population, T1 and T2 transitional B cells within the CD93^+^ population, and FO and MZ B cells within the CD93^−^ population. (**E**–**G**) Frequency of the indicated spleen B cell subsets, expressed as mean ± SD, *n* = 4–5, *P < 0.05, **P < 0.01 by 2-tailed *t* test.

One of the most prominent features of hematological issues of ZTTK syndrome is low immunoglobulin level, which was recapitulated by our *Son^+/–^* mice (Figure 3H). To address whether this is attributed to impaired B cell development in *Son^+/–^* mice, we first analyzed a series of B cell subsets in the bone marrow (Hardy fractions; ref. 36) as well as the spleen (Figure 6A and Supplemental Figure 9, C and D). While bone marrow B cells are mostly intact and not altered in *Son^+/–^* mice, IgD expression was slightly reduced within the CD43^+^ late-state developing B cell population, resulting in increased small Pre-B fraction (fraction D) and decreased recirculating B fraction (fraction F) (Figure 6B and Supplemental Figure 9, C and D). The most interesting alteration was observed in CD93 (AA4.1) (37) analysis in the spleen (Figure 6C). We found that CD93^+^ immature transitional B cells were significantly increased, and in contrast, CD93*^–^* naive mature B cells were decreased in the *Son^+/–^* mouse spleen (Figure 6, D and E).

We further analyzed T1 and T2 cells (38) using the IgM and IgD expression levels and found that IgD^lo^ T1 cells were increased while IgD^hi^ T2 cells were decreased (Figure 6, D and F). These results indicate that within transitional B cells, the developing process from the “T1” subset to the “T2” subset is hindered, resulting in a blockage that prevents transitional B cells from progressing to naive mature B cells. Further analyses of the CD93^−^ naive B cell population demonstrated a reduced fraction of follicular (FO) B cells (CD23^+^CD21^intermediate^IgM^lo^IgD^hi^) and an increased marginal zone (MZ) B cell fraction (CD23^−^CD21^hi^IgM^hi^IgD^lo^) in *Son^+/–^* mice (Figure 6, D and G). These findings revealed substantial perturbations in the normal early B cell maturation processes within the spleen of *Son^+/–^* mice, which are likely contributing factors to the observed low levels of immunoglobulins in both *Son^+/–^* mice and patients with ZTTK syndrome.

### Single-cell profiling and transcriptional state–based clusters of HSPCs reveal substantial decreases of lymphoid/B cell lineage clusters in Son^+/–^ mice.

**Figure 7:**
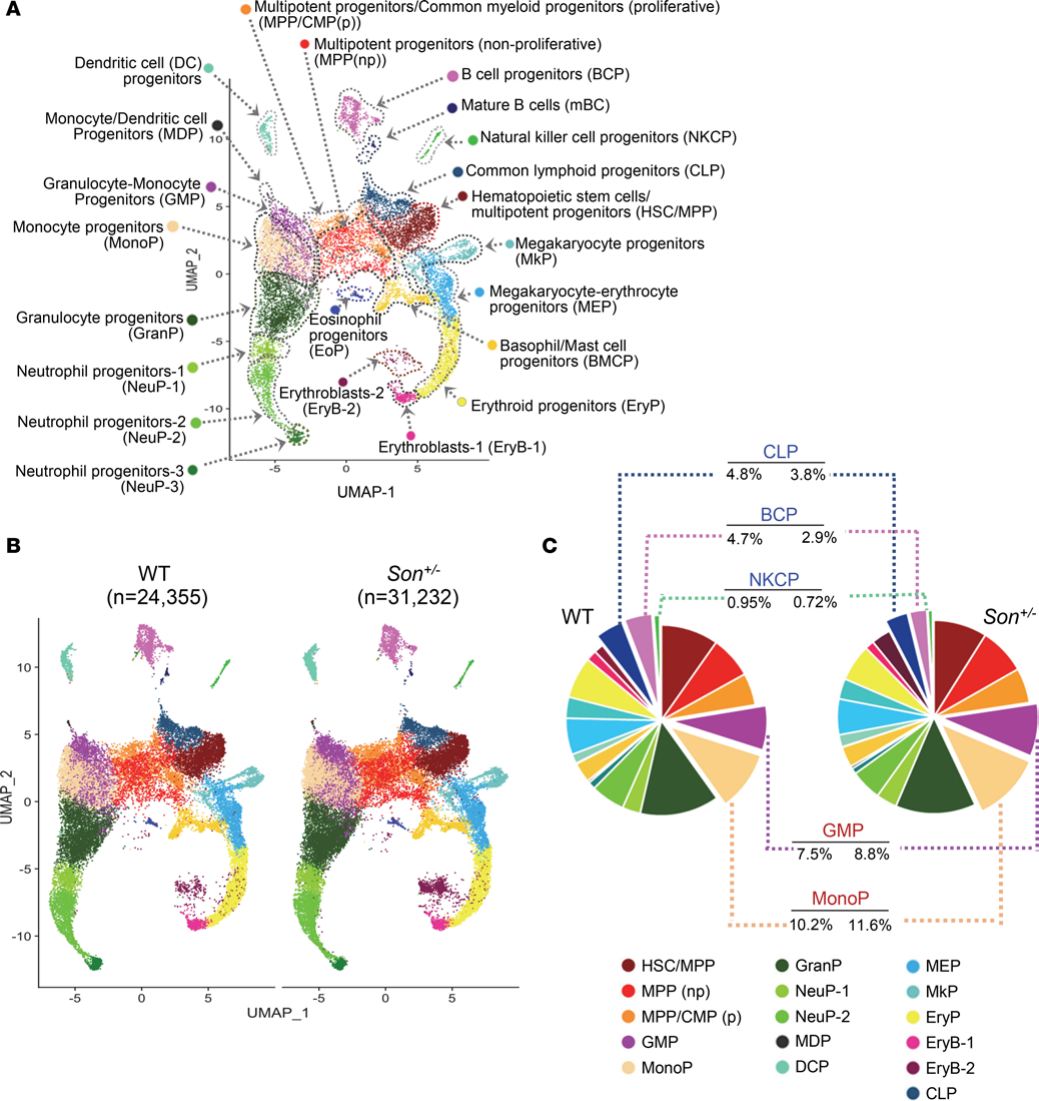
scRNA-Seq and transcriptional state–based clustering of HSPCs reveal significant decreases in lymphoid lineage clusters in *Son*^+/–^ mice. (**A**) Uniform manifold approximation and projection (UMAP) plot showing 22 cell clusters identified from scRNA-Seq of mouse bone marrow LK cells. Cell clusters are annotated based on the enriched genes and cell cycle status. (**B**) UMAP plots showing the 22 clusters, as described in **A**, identified in the bone marrow LK cells from WT and *Son^+/–^* mice. (**C**) Pie chart illustrating the color-coded cell populations of the average percentages of each cell cluster within total annotated cells. Average percentage values were from separate analyses of 2 WT mice (12,173 and 12,182 total annotated cells) and 2 *Son^+/–^* mice (11,764 and 19,468 total annotated cells). Clusters that showed a consistent increase (GMP and MonoP) or a decrease (CLP, BCP, and NKCP) in 2 *Son^+/–^* mice compared with 2 WT are indicated.

To obtain a more comprehensive understanding of the changes within HSPCs at the molecular level, we employed single-cell RNA-sequencing (scRNA-Seq) using both WT and *Son^+/–^* bone marrow HSPCs isolated and sorted based on their LK cell markers (24,539 total LK cells from 2 WT mice; 31,521 total LK cells from 2 *Son^+/–^* mice; Supplemental Figure 10, A and B). By conducting an in-depth analysis of the transcriptional states (Supplemental Data 1) and cell cycle phases of individual cells (Supplemental Figure 10C), we identified 22 distinct clusters that represent the various hematopoietic stem and progenitor populations and their lineage trajectories (Figure 7A).

While the HSPCs of both WT and *Son^+/–^* mice shared similar cluster maps (Figure 7B), we observed several notable differences in the sizes of certain clusters. We found that *Son^+/–^* mice had an increased proportion of GMPs and monocyte progenitors (MonoPs) (Figure 7C). Of particular interest, we observed substantial reductions in the size of clusters containing lymphoid lineage cells in *Son^+/–^* mice. These clusters include common lymphoid progenitors (CLPs), B cell progenitors (BCPs), and natural killer cell progenitors (NKCPs) (Figure 7C). Among these, BCPs exhibited the most noticeable reduction. These transcriptome-based clusters revealed that consistent with our data from surface marker–based analysis, *Son* haploinsufficiency leads to a reduction in lymphoid lineage–committed progenitor cells and an increase in early-stage myeloid lineage progenitors.

### Altered gene expression patterns were identified in several HSPC clusters in Son^+/–^ mice, including decreased expression of lymphoid/B cell lineage development genes and aberrant upregulation of erythroid lineage–promoting genes.

**Figure 8:**
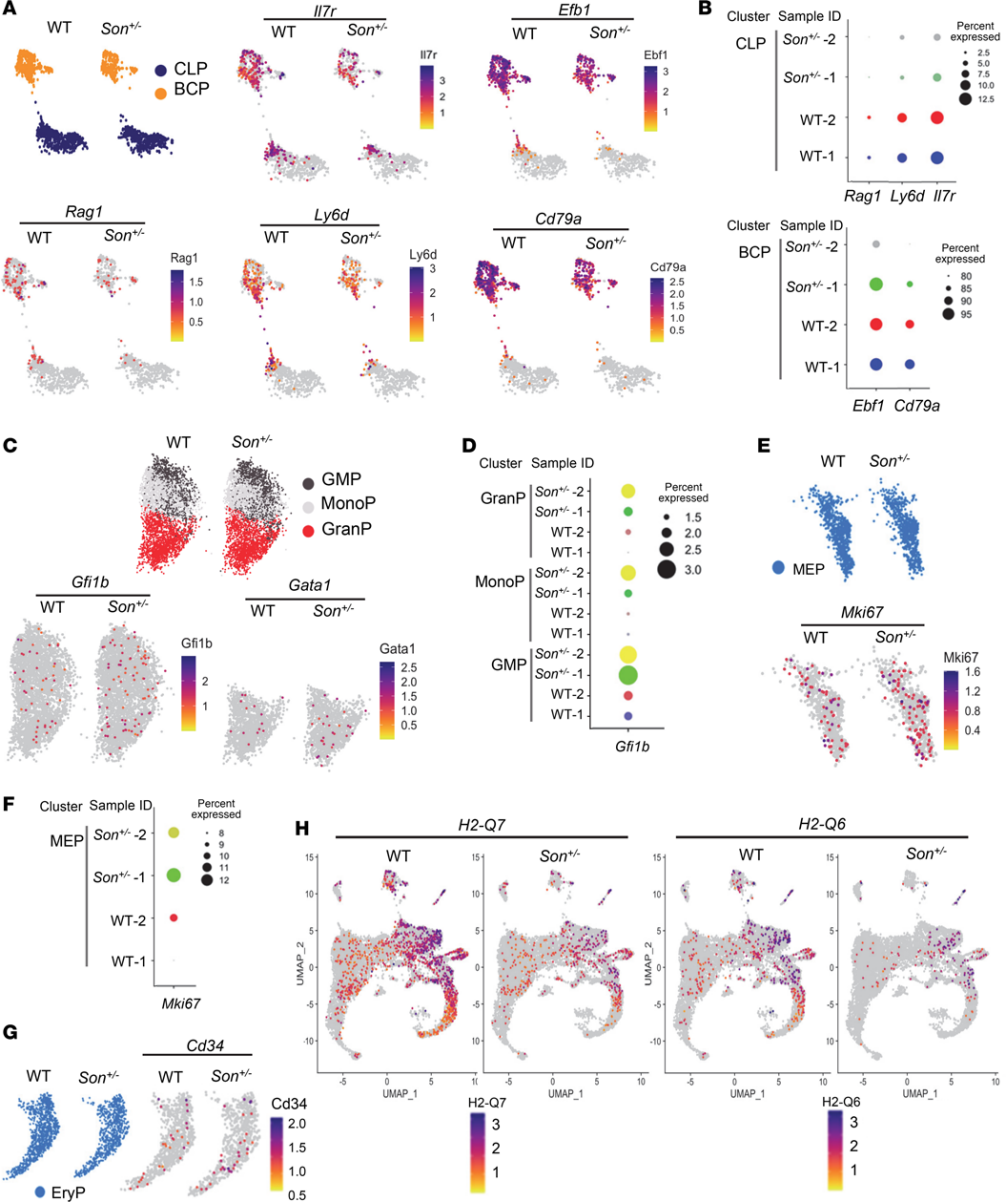
Differential gene expression analysis in hematopoietic progenitor clusters identified altered expression of genes critical for lymphoid/B cell lineage development and aberrant activation of myeloid lineage–regulating genes in *Son^+/–^* mice. (**A**) Feature plots showing the common lymphoid progenitor (CLP) and B cell progenitor (BCP) clusters from WT and *Son^+/–^* mice and the expression distribution of indicated genes critical for lymphoid lineage specification and B cell development. (**B**) Dot plots visualizing the percentage of the cells expressing the indicated genes. The size of the dot encodes the percentage of cells within the cluster. (**C** and **D**) Feature plots (**C**) and dot plots (**D**) showing the granulocyte/monocyte progenitor (GMP), monocyte progenitor (MonoP), and granulocyte progenitor (GranP) from WT and *Son^+/–^* mice and the expression of the erythroid lineage transcription factors. (**E** and **F**) Feature plots (**E**) and dot plots (**F**) of megakaryocyte/erythroid progenitor (MEP) expressing MKi67. (**G**) Feature plots showing Cd34 expression in the erythroid progenitor (EryP) cluster. (**H**) Feature plots showing the expression levels of *H2-Q7* and *H2-Q6* genes in all clusters from WT and *Son^+/–^* mice. (**A**, **C**, **E**, **G**, and **H**) Expression levels for each cell are color-coded as indicated in the heatmap legend.

To investigate the impact of *Son* haploinsufficiency within specific HSPC populations, we analyzed differentially expressed genes (DEGs) in each cluster. Notably, we observed a notable downregulation of critical genes related to lymphoid lineage and B cell lineage development in the CLP and BCP clusters of *Son^+/–^* mice compared with those in WT mice (Figure 8, A and B). These genes include *Il7r* (essential for lymphoid lineage cell development), *Ebf1* (a key transcription factor for B cell lineage commitment), *Ly6d* (required for early-stage B cell specification), *Rag1* (critical for immunoglobulin gene rearrangement), and *Cd79a* (a critical component of B cell receptor signaling) (39–44). For these genes, we observed not only decreased expression levels per cell (Figure 8A) but also a decreased percentage of the cells expressing the assigned gene within a cluster (Figure 8B).

Interestingly, we observed abnormal upregulation of critical erythroid lineage transcription factor genes, such as *Gfi1b* (45) and *Gata1* (46), in the granulocyte and/or MonoP populations of *Son^+/–^* mice (Figure 8, C and D), suggesting that erythroid genes were inappropriately turned on in the granulocyte/monocyte lineage. In the MEP cluster, we detected upregulation of the *Mki67* gene (Figure 8, E and F), which encodes Ki-67, a marker of proliferation and active cell cycle (47). Additionally, we observed upregulation of *Cd34*, a maker enriched in immature progenitors (48), in the *Son^+/–^* erythroid progenitor cluster (EryP) (Figure 8G).

In addition to the changes in lineage-specific gene expression, our DEG analysis revealed a marked downregulation of *H2-Q6* and *H2-Q7*, the nonclassical MHC molecules functioning in antigen presentation and regulation of immune responses (49, 50), in multiple cell types in *Son^+/–^* HSPCs, including the HSC/MPP cluster (Figure 8H).

Taken together, our analysis, based on transcription state–based clustering and gene expression, highlights the molecular mechanisms underlying decreased lymphoid lineage progenitors, defective B cell development, and increased early-stage myeloid progenitors with erythroid lineage potential in *Son^+/–^* hematopoietic cells.

## Discussion

Given recent discoveries on the pathogenic effects of compromised SON function in ZTTK syndrome, as well as the diverse cellular functions of SON in RNA splicing, transcription, and nuclear speckle organization, there is a pressing need for appropriate model organisms with disrupted SON expression. In this study, we created a genetically engineered mouse line of *Son* deletion and demonstrated that *Son* is indispensable for embryo development. Using the *Son^+/–^* mouse model, we identified the importance of the full gene expression of *Son* in various organ development and hematopoiesis, which explains multiple phenotypes found in human ZTTK syndrome. Furthermore, our scRNA-Seq data provide a comprehensive view of the hematopoietic stem and progenitor landscape in *Son^+/–^* mice and highlights the potential molecular mechanisms underlying the observed phenotypic alterations.

We emphasize the value of our mouse model in both biological and clinical aspects. First, our mouse model is a useful tool for biological research to study SON’s role in organ development and cell type–specific gene expression. Partially because of its large gene size (~7.3 kb for the coding sequence) and protein size (consisting of 2,426 amino acids), cellular functions of SON have not been under extensive research. In addition, the sequence analysis predicted that SON contains an intrinsically disorganized region lacking any fixed structure (23), which poses challenges for structure-based functional studies. Although our group has reported that SON mediates efficient RNA splicing and represses MLL complex–associated transcriptional initiation, there could be many unknown biological functions of SON that remain unexplored. Recent studies have demonstrated that SON, together with SRRM2, forms a core of nuclear speckles to regulate nuclear speckle integrity (23) and is involved in boosting p53-mediated gene expression (24). Interestingly, SON is a critical factor in regulating the 12-hour rhythm of nuclear speckle phase separation dynamics (25). Considering these multiple roles of SON in cellular functions, a mouse model of *Son* knockout and haploinsufficiency is needed. In this study, we present the newly created mouse lines, *Son^fl/fl^* and *Son^+/–^*, which will serve as invaluable resources to study SON function in various organs as well as at different developmental stages. We show that homozygous knockout of *Son* (*Son^–/–^*) caused embryonic lethality, defining the indispensable roles of *Son* in embryo development. In addition, using the *Son^+/–^* mouse model and the SON antibody we generated, we present the Son protein expression pattern in the whole body of developing embryos and several adult tissues and verify the specificity of our findings by comparing *Son^+/–^* embryos/mice with WT controls. These analyses raise many outstanding questions regarding SON’s roles in different tissues/organs. Given that SON has multiple roles in regulating RNA splicing, transcription, and nuclear speckle dynamics, the molecular mechanisms underlying each phenotype should be thoroughly examined in future research through comprehensive analyses of the transcriptome and RNA splicing. Identifying cell type–specific and tissue-specific target genes will be critical for us to understand the molecular mechanisms of each clinically relevant phenotype observed in our mouse model. For example, investigating how *Son* haploinsufficiency affects osteoblast generation and osteoclast activities, and whether any crosstalk between the bone and the marrow compartment is altered by *Son* haploinsufficiency, will provide further insights into the mechanism underlying the skeletal phenotype. In addition, this knowledge will serve as the basis for developing potential strategies to prevent or alleviate the symptoms.

The other aspect and more exciting point we emphasize here is the value and usefulness of the *Son^+/–^* mouse model for clinical research on human ZTTK syndrome. The pathogenic effect of *SON* LoF variants on causing this complex human genetic disease was first reported in 2016 by our group and others. These findings made major advances in clinical diagnosis and led to incredible family effort and participation, which launched an official patient/family-initiated foundation, the ZTTK SON-Shine Foundation. However, the roadblock that clinical practice for rare diseases is facing is the unclear definition of clinical symptoms because of low prevalence and lack of attention from research communities. This causes extreme frustration to families and difficulties for clinicians to guide the patients and families for follow-up care. Our *Son^+/–^* mouse model recapitulates many of the clinical features of human ZTTK syndrome. Although more extensive studies will be required to fully delineate underlying molecular mechanisms, we present that our mouse model could serve as a useful tool to study each of these clinically significant features and develop therapeutics to improve or alleviate clinical symptoms.

Besides characterizing the clinical features mentioned above, we highlighted hematopoietic features of *Son^+/–^* mice, which are closely linked to the symptoms found in ZTTK syndrome, including leukopenia, macrocytosis, and thrombocytopenia, and identified the altered hematopoietic lineage differentiation in *Son^+/–^* mice. We identified several critical changes in hematopoiesis caused by *Son* haploinsufficiency, including increased myeloid lineage–biased MPPs, especially MPP2, and decreased MPP4 in early-stage hematopoiesis. This finding is particularly interesting because the expansion of myeloid lineage and reduction of lymphoid lineage early-stage hematopoietic progenitor cells are typical features found in aged HSCs and are associated with various hematopoietic diseases, including myelodysplastic syndrome (MDS) and myeloproliferative neoplasm (MPN) (51–56). Our study provides a significant guideline for clinical practice for ZTTK syndrome with hematopoietic involvement and suggests the need for monitoring myeloid expansion, including MDS and MPN, in these patients.

Interestingly, at the level of lineage-committed progenitor cells, we found an increase in the pre–CFU-E population, which are erythroid lineage–committed precursors preceding CFU-E, in the phenotypic analysis of *Son^+/–^* mouse bone marrow. The increased pre–CFU-E could be due to increased erythroid fate specification from the progenitors with megakaryocytic/erythroid lineage bipotential (MEP or pre-MegE) or a blockage of erythroid differentiation. Our scRNA-Seq data, showing Mki67 upregulation in the MEP cluster and Cd34 upregulation in the EryP cluster, suggest that it is likely that both erythroid-favored lineage specification and impaired erythroid differentiation contribute to the pre–CFU-E increase.

It is worth noting that our scRNA-Seq data also indicate an overall increased size of the late-stage erythroblast cluster in *Son^+/–^* mouse bone marrow (EryB-2; Figure 7) when we combined the cells from 2 mice. However, we did not present it as a main point in this report due to the variation observed between the 2 *Son^+/–^* mice. Interestingly, the Blood Spot database (www.bloodspot.eu) showed that the erythroid differentiation process in humans accompanies a continuously high level of SON expression with a further transient increase in CD71^+^GlyA^−^ erythroblasts (Supplemental Figure 11), which supports the notion that full gene expression of *Son* is critical for proper erythroid differentiation. A recent study has demonstrated a connection between rapid cell cycle and increased MCV (57). Further analyses of erythroid lineage clusters and the underlying mechanisms, such as gene expression and cell cycle speed, would reveal how *Son* haploinsufficiency affects erythroid lineage differentiation and causes MCV increases in human ZTTK syndrome and *Son^+/–^* mice.

In our bone marrow LK cell scRNA-Seq, the most significant impact of *Son* haploinsufficiency on hematopoietic gene expression was the downregulation of genes critical for lymphoid development and B cell lineage development, such as *Il7r*, *Ebf1*, *Rag1*, *Ly6d*, and *Cd79a*, in the transcriptionally defined CLP and BCP clusters. Together with surface marker–based phenotypic analysis showing impaired B cell maturation, our data suggest that the normal level of *Son* is required to express sufficient levels of genes directing lymphoid lineage specification and B cell development. Further investigations on terminal differentiation processes of each lineage and the origin of lineage bias will enable us to understand more about SON’s role in different hematopoietic lineages and how *SON* haploinsufficiency alters hematopoiesis in ZTTK syndrome. Our study supports the notion that CBC abnormalities and low levels of immunoglobulins should be considered as direct outcomes of *SON* LoF, and these symptoms should be incorporated in the diagnosis process.

About 95% of rare diseases do not have treatments because of the lack of research on these diseases. Our attention to rare disease research is critical to improve the quality of patients’ lives. Our data presented here will elicit future research on ZTTK syndrome for individuals, particularly children, with this rare disease. We anticipate our study to be a key reference for the rare disease community and promote rare disease research.

## Methods

### Sex as a biological variable.

Our study examined male and female mice. Sex-dimorphic effects on offspring genetic outcome are reported. For all other phenotypes, similar findings are reported for both sexes.

### Generation of Son^fl/fl^ mice.

*Son^fl/fl^* mice were generated by the CRISPR/Cas9-based Extreme Genome Editing system (Biocytogen). Briefly, 2 single guide RNAs (sgRNAs) were designed to target the nonconserved intron regions flanking exon 2 (based on the Ensembl transcript ID ENSMUST00000114037.8; National Center for Biotechnology RefSeq NM_178880.4) within the mouse *Son* gene (NC_000082.6) located on mouse chromosome 16. The selected sgRNA and Cas9 mRNA were coinjected into C57BL/6 mouse zygotes together with the targeting vector containing homologous arms and the *loxP* element. The zygotes were then transplanted into pseudopregnant females to generate founder mice bearing the floxed *Son* allele. These mice were backcrossed to WT C57BL/6J mice (Jackson Laboratory; Strain 000664) for at least 4 generations, followed by intercrossing to generate mice homozygous for the floxed allele (*Son^fl/fl^*).

### Generation of constitutive Son^+/–^ mice.

*Son^fl/fl^* mice were crossed to β-actin-cre (*ACTB-cre*) mice [Jackson Laboratory, Strain 019099; B6N.FVB-*Tmem163^Tg(ACTB-cre)2Mrt^*/CjDswJ] to obtain *ACTB-Cre^+^ Son^fl/wt^* mice. To obtain mice with heterozygous *Son* deletion in germline, *ACTB-Cre^+^ Son^fl/fl^* mice were backcrossed to WT C57BL/6J mice, and offspring with excision of the floxed sequence within the *Son* gene in the absence of the *Cre* transgene were identified. The selected offspring were further backcrossed to WT C56BL/6J mice for more than 6 generations to ensure stable germline transmission of the *Son* allele with excision of deletion. The resulting mice have a constitutive, heterozygous deletion of *Son* exon 2 from germline (*Son^+/–^*) without Cre expression, which causes a premature stop codon after encoding 9 amino acids in exon 3.

### IHC staining of embryos.

Formalin-fixed, paraffin-embedded embryo sections were heated (60°C for 30 minutes) in an oven, deparaffinized, and hydrated in xylene and 100%, 95%, 80%, 70% ethanol, then in distilled H_2_O. After unmasking antigens in boiling citrate buffer (MilliporeSigma, C9999) with pH 6.0 for 40 minutes, the sections were incubated in 3% hydrogen peroxide for 10 minutes, rinsed with water, and blocked with 5% goat serum diluted in Tris-buffered saline with 0.1% Tween 20 (TBST) for 30 minutes at room temperature (RT). Then, sections were incubated with the primary antibody, anti-SON generated against SON amino acids 77–84 (22), overnight at 4°C. After washing with TBST (0.1%), sections were incubated in a secondary antibody (anti-rabbit; Abcam AB205718) for 30 minutes at RT and then with DAB (Thermo Fisher Scientific PI34002) for 30 seconds. After incubation with hematoxylin (Vector Laboratories, H3404) for 1 minute, slides were washed in distilled H_2_O, dipped in 0.3% ammonia water for 2 seconds, and dehydrated in 70%, 80%, 95%, 100% ethanol and xylene. Images were taken using the Lionheart LX automated microscope (Agilent).

### Western blot analysis.

Proteins from mouse tissue were extracted with 1× RIPA buffer (150 mM NaCl/1% sodium deoxycholate/1 mM EDTA/0.1% SDS/50 mM Tris pH 8/1% Triton X-100); Pierce Protease Inhibitor, EDTA-Free (Thermo Fisher Scientific, A32965); and Pierce Phosphatase inhibitor (Thermo Fisher Scientific, A32957). Samples were centrifuged at 13,000*g* for 30 minutes at 4°C, and the supernatant protein samples were separated on 7% SDS-PAGE and transferred onto the PVDF membrane. Membranes were blocked in 5% BSA in 1× TBST (0.2%) for 1 hour at RT and incubated with the respective primary antibodies overnight at 4°C. Antibodies that were used included anti-SON (22) and β-actin (Cell Signaling Technology, 3700). Blots were washed in 1× TBST (0.2%) at RT and incubated with the secondary antibody for 2 hours on a rotator at RT, followed by three 10 minutes/wash in 1× TBST (0.2%) at RT. Chemiluminescence detection was performed with the x-ray film (Alkali Scientific) and x-ray film processor (Konica).

### Alizarin red and Alcian blue staining of skeletons.

Neonatal mouse pups (day 1) were euthanized and scalded in water (65°C) for 30 seconds, and internal organs were removed, followed by fixing in 95% ethanol overnight and incubating in acetone overnight. The fixed samples were stained with Alcian blue overnight at RT and then destained by incubation in 95% ethanol overnight. Then, the samples were incubated in 1% KOH for 1 hour at RT followed by incubation in alizarin overnight at 4°C. Then, samples were incubated in 50% glycerol:50% of 1% KOH solution at RT until the specimen became clear. Skeletons were then transferred to 100% glycerol until images were taken.

### μCT.

Mouse full-body scans were performed under isoflurane in oxygen anesthesia and prospectively gated for a single respiration phase using the following settings: 50 kVp, 0.24 mA, 20 ms exposure, 20 μm voxel size, using the U-CT^UHR^ μCT scanner (MiLabs). Scans were reconstructed using vendor software. For bone scans, femurs were isolated from 6-week-old female mice. Then skeletal muscles were removed and fixed in formalin for 48 hours followed by storage in 70% ethanol. Femurs were placed in a 12 mm diameter scanning holder and imaged with the following settings: 70 kVp, 114 μA with an integration time of 200 millisecond, and 500 projections per 180°, 12 μm voxel size, using the μCT40 desktop cone-beam scanner (Scanco Medical AG, using μCT Tomography v6.4-2). Scans were reconstructed into 2D slices, and all slices were analyzed using the μCT Evaluation Program (v6.5-2, Scanco Medical). 3D reconstruction was performed using μCT Ray v4.2.

### CBC and measurement of immunoglobulins.

Peripheral blood counts were obtained by using heparin-coated microvettes (Sarstedt), and heparinized blood was subjected to analysis using Hemavet 950FS for CBCs. IgM, IgG, and IgA titers in mouse plasma were measured using the ELISA kits from Bethyl Laboratories (IgM catalog E99-101; IgG catalog E99-131; IgA catalog E99-103) according to the manufacturer’s instructions. The ELISA results were assessed with the BioTek Synergy H1 microplate reader (Agilent).

### Mouse behavior assessments.

The open field test was conducted in a square Plexiglass box for 10 minutes, and the animal was tracked with an overhead camera. The automated tracking system (EthoVision) analyzed parameters such as distance moved, velocity, acceleration, and time spent in predefined zones. The Y-maze spontaneous alternation test was used to assess the willingness of mice to explore new areas. The symmetrical Y-shaped Plexiglass maze had a white bottom and walls, and each mouse was allowed to move freely for 5 minutes. The observer recorded the time spent by the subject in each arm and manually recorded spontaneous alternations to ensure that the mouse explored a new arm each time it returned to the center of the maze. The percentage of spontaneous alternation was calculated as the number of consecutive entries into all 3 arms divided by the number of possible alternations. A novel object recognition test was conducted over 3 consecutive days. On the first day, mice explored the open field without any objects for 5 minutes. On the second day, 2 green-colored wooden cubes were introduced, and mice were allowed to explore for 5 minutes. On the third day, a novel object, an orange-colored wooden cylinder, was introduced along with a familiar object, a green-colored wooden cube. The mouse nose touching or pointing within 12 cm of the object was considered positive exploration, and the time spent exploring each object was manually recorded. The discrimination index was calculated using the ratio of time spent exploring the novel object to the time spent exploring both objects.

### Flow cytometry.

Bone marrow cells were isolated from the tibia and femur by flushing a 25G syringe with 1× PBS containing 2% FBS. The RBCs were removed using ACK lysis buffer before antibody incubation. For fetal liver cell analysis, timed pregnancies were performed, and embryos were harvested at different days postconception into 1× PBS supplemented with 2% FBS. Fetal livers were isolated and dissociated using a pipette in fetal liver juice, which consisted of 2% FBS, 2.5 mM EDTA, and 10 mM glucose in PBS. The cells were then counted and incubated with antibodies listed in Supplemental Table 2. The cell populations were analyzed using BD Biosciences FACSCanto II, BD Biosciences LSRII, and BD Biosciences FACSymphony A5 flow cytometers.

### scRNA-Seq and data analysis.

The Lin^–^c-kit^+^ population containing HSPCs was sorted from dissociated mouse bone marrow cells by flow cytometry. Sorted single-cell suspension and barcoded gel beads and oil (10x Genomics Chromium Next GEM Single Cell 3′ Kit v3.1 PN 1000268) were loaded into 10x Genomics Chromium Single Cell Chip to capture single cells into nanoliter-scale oil droplets by 10x Chromium X Controller and generate Gel Bead-In-EMulsions (GEMs). cDNA from single cells was synthesized and barcoded by incubation of the GEMs in a thermocycler machine and then purified from GEMs by DynaBeads (10x Genomics PN 2000048). cDNA was preamplified by PCR to generate sufficient mass for sequencing library construction. The single-cell cDNA libraries were constructed by following 10x Genomics 3′ NextGem 3.1 version kit instructions. The final constructed 3′-biased single-cell libraries were sequenced by the Illumina NovaSeq 6000 machine, targeting 50,000 read pairs/cell.

The Cell Ranger 7.1.0 (10x Genomics) pipeline was used to align the raw sequencing data to the mm10 mouse reference genome and count the expressed transcripts. Seurat (v4.3.0) was used to analyze and visualize the processed files. Detailed methods used for data analysis are described in Supplemental Methods.

### Statistics.

Data are presented as ± SD. *P* values considered significant are presented below each figure. Statistical significance was determined by 2-tailed *t* test and log-rank (Kaplan-Meier). Statistical analyses were performed using GraphPad Prism.

### Study approval.

All the procedures of breeding and experiments using mice were approved by and performed following the guidelines of the University of Alabama in Birmingham Institutional Animal Care and Use Committee.

### Data availability.

The scRNA-Seq data have been deposited in the National Center for Biotechnology Information’s Gene Expression Omnibus under the accession number GSE252833. Values for all data points in graphs are available in the Supporting Data Values file.

## Author contributions

LV, JHK, STSL, and EYEA conceptualized the project. LV, BP, MM, HL, AE, ATD, JHK, KJ, JMM, RAB, AGS, RSW, BEH, STSL, and EYEA designed the experiments, developed the methodology, and analyzed the data. LV, BP, MM, HL, AE, ATD, JHK, KJ, JMM, LG, HL, HGK, JPS, SNL, DS, and BEH performed the experiments and collected data. MM and EYEA analyzed scRNA-Seq data, and JBF evaluated kidney H&E. LV, BP, and EYEA wrote the original draft of the manuscript with input from all authors. LV, BP, MM, AE, CAH, SD, LG, BEH, STSL, and EYEA reviewed and edited the manuscript. BP, MM, STSL, and EYEA revised the manuscript. EYEA supervised the overall study. Equal-contribution author order was determined by the authors themselves.

## Supplementary Material

Supplemental data

Supplemental data set 1

Unedited blot and gel images

Supporting data values

## Version 1 01/30/2024

In-Press Preview

## Version 2 03/08/2024

Electronic publication
