# Supplementary material for: A mouse model of Zhu-Tokita-Takenouchi-Kim syndrome reveals indispensable SON functions in organ development and hematopoiesis
Source: JCI Insight. 2024 Jan 30;9(5):e175053. doi: 10.1172/jci.insight.175053 (PMC10972584; doi:10.1172/jci.insight.175053)
Supplement: Supplemental data [file jciinsight-9-175053-s065.pdf]

A mouse model of Zhu-Tokita-Takenouchi-Kim syndrome reveals indispensable SON functions in organ development and hematopoiesis

Lana Vukadin[#], Bohye Park[#], Mostafa Mohamed[#], Huashi Li, Amr Elkholy, Alex Torrelli-Diljohn, Jung-Hyun Kim, Kyuho Jeong, James M Murphy, Caitlin A. Harvey, Sophia Dunlap, Leah Gehrs, Hanna Lee, Hyung-Gyoon Kim, Jay Prakash Sah, Seth N. Lee, Denise Stanford, Robert A. Barrington, Jeremy B. Foote, Anna G. Sorace, Robert S. Welner, Blake E. Hildreth III, Ssang-Taek Steve Lim, and Eun-Young Erin Ahn

Supplemental Materials

Supplemental Figures 1 – 11

Supplemental Tables 1 and 2

Supplemental Methods

Supplemental Figures

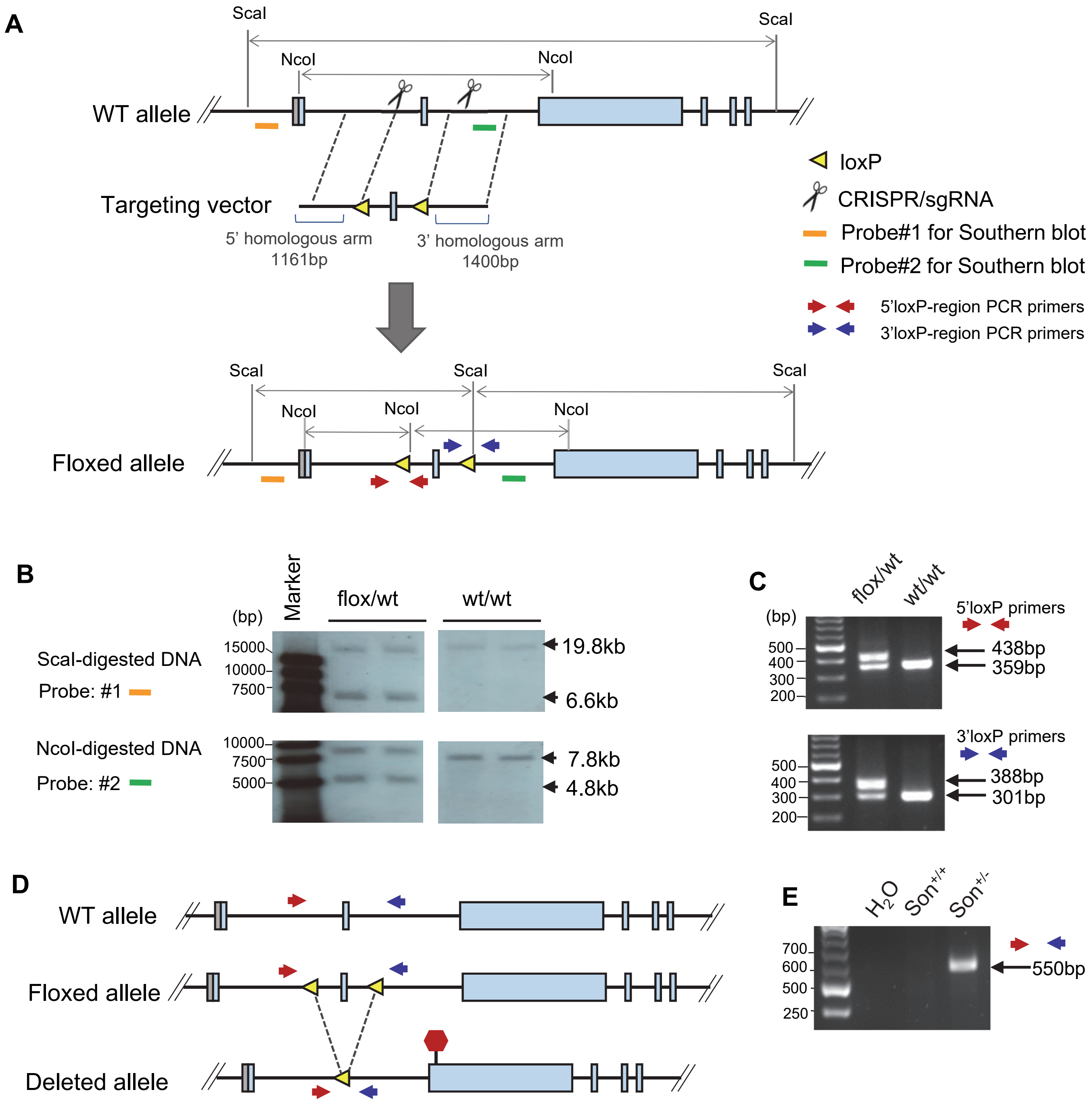

Supplemental Figure 1. Generation of *Son*-floxed mice and *Son*$^{+/-}$ mice. (**A**) Strategy for homologous recombination at the mouse *Son* gene locus and the locations of the probes and primers used for Southern blot and PCR shown in panels B, C, and E. This design is based on ENSMUST00000114037.9 (mouse *Son* transcript-202 in Ensembl; NM_178880.4). (**B**) Southern blot analysis of ScaI-digested and NcoI-digested genomic DNA from tail snips of F1 mice that are heterozygous for the floxed allele, along with wild type control. (**C**) PCR genotyping to detect the floxed allele. (**D**) Schematics comparing the WT, floxed, and deleted allele and the location of a premature termination codon (red hexagon) formed upon deletion of the floxed region. (**E**) PCR genotyping to detect the deleted allele.

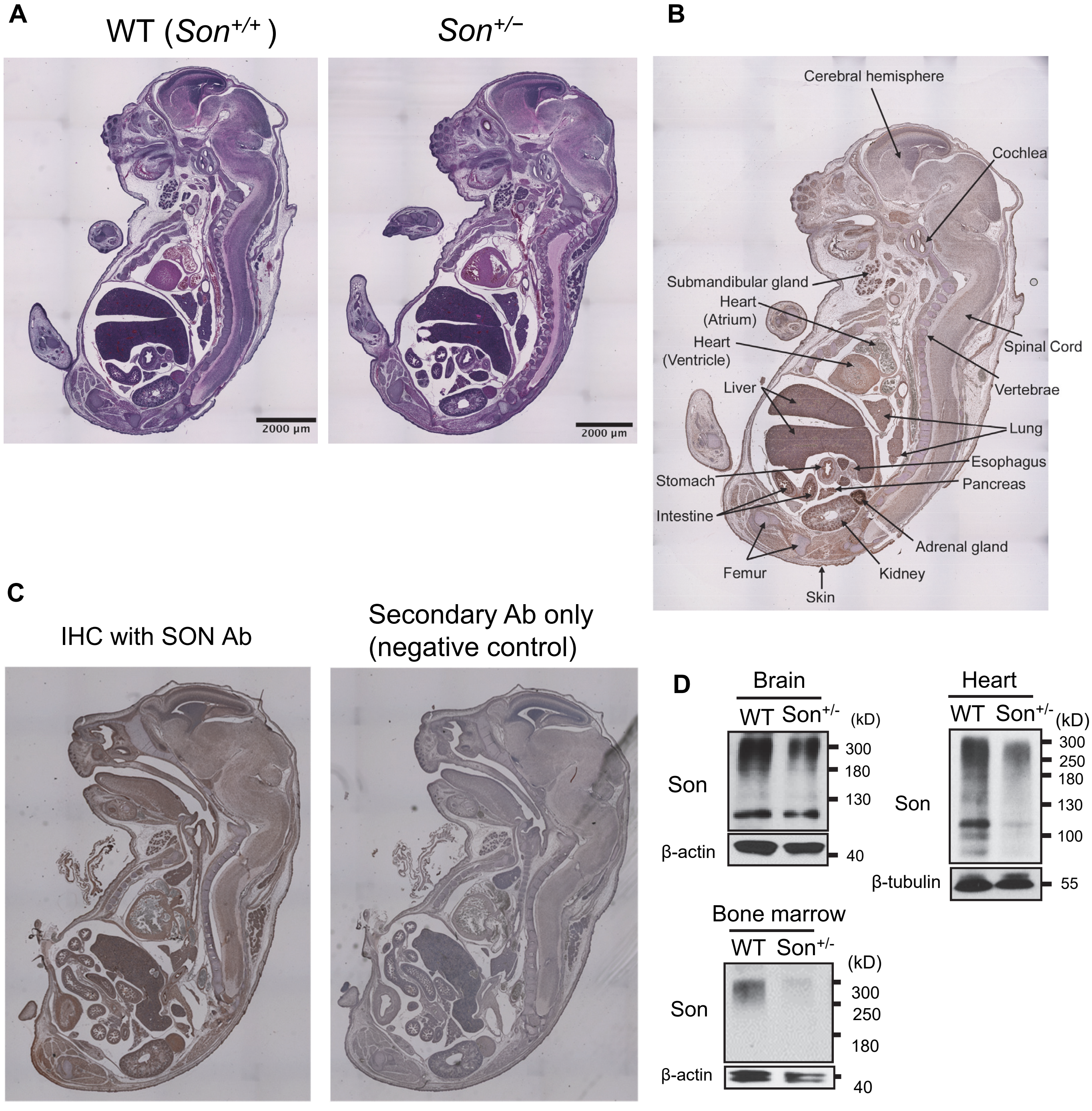

Supplemental Figure 2. Analysis of Son expression in embryos and adult tissues of *Son*$^{+/-}$ mice. (**A**) H&E staining of the sagittal section of WT and *Son*$^{+/-}$ embryos (E16), showing no significant defects in forming fetal organs in the *Son*$^{+/-}$ embryos. (**B**) An image of an E16 WT embryo and annotations of tissue/organs. (**C**) IHC staining without primary antibody (secondary antibody only) is completely devoid of immunopositivity, indicating specificity of brown staining detected by Son antibody (Ab). (**D**) The expression of Son in the indicated organs of WT and *Son*$^{+/-}$ adult mice was analyzed by Western blot.

Age		Male WT	Male Son$^{+/-}$	Male p	Female WT	Female Son$^{+/-}$	Female p
4 weeks	Weight (g)	18.8 ± 0.8	16.3 ± 2.1	$p < 0.01$	16.7 ± 0.6	13.0 ± 1.0	$p < 0.01$
	Length (cm)	8.5 ± 0.1	8.0 ± 0.0	$p < 0.001$	8.3 ± 0.3	8.0 ± 0.0	$p = 0.1161$
8 weeks	Weight (g)	25.0 ± 0.7	21.5 ± 0.8	$p < 0.001$	20.7 ± 1.2	16.3 ± 0.6	$p < 0.01$
	Length (cm)	9.7 ± 0.2	9.0 ± 0.1	$p < 0.01$	9.1 ± 0.2	8.7 ± 0.1	$p < 0.05$
12 weeks	Weight (g)	29.6 ± 0.5	23.0 ± 1.7	$p < 0.001$	24.0 ± 1.7	18.3 ± 0.6	$p < 0.01$
	Length (cm)	10.0 ± 0.0	9.4 ± 0.3	$p < 0.05$	9.5 ± 0.5	9.0 ± 0.0	$p = 0.1583$
21 weeks	Weight (g)	39.8 ± 1.3	27.5 ± 2.9	$p < 0.001$	32.0 ± 3.6	20.3 ± 1.5	$p < 0.01$
	Length (cm)	10.7 ± 0.1	9.7 ± 0.1	$p < 0.001$	10.2 ± 0.1	18.9 ± 0.1	$p < 0.001$
30 weeks	Weight (g)	44.3 ± 2.3	27.5 ± 2.3	$p < 0.001$	35.2 ± 4.4	20.9 ± 1.7	$p < 0.01$
	Length (cm)	10.8 ± 0.2	9.8 ± 0.2	$p < 0.001$	10.2 ± 0.1	9.2 ± 0.3	$p < 0.01$
40 weeks	Weight (g)	47.7 ± 3.3	30.0 ± 1.7	$p < 0.001$	37.8 ± 5.5	21.6 ± 0.9	$p < 0.01$
	Length (cm)	11.0 ± 0.1	9.7 ± 0.1	$p < 0.001$	10.4 ± 0.1	9.1 ± 0.1	$p < 0.001$
60 weeks	Weight (g)	53.0 ± 2.5	30.8 ± 1.1	$p < 0.001$	39.3 ± 4.3	22.8 ± 1.3	$p < 0.05$
	Length (cm)	10.6 ± 0.1	9.5 ± 0.2	$p < 0.001$	10.6 ± 0.1	9.3 ± 0.3	$p < 0.01$
75 weeks							

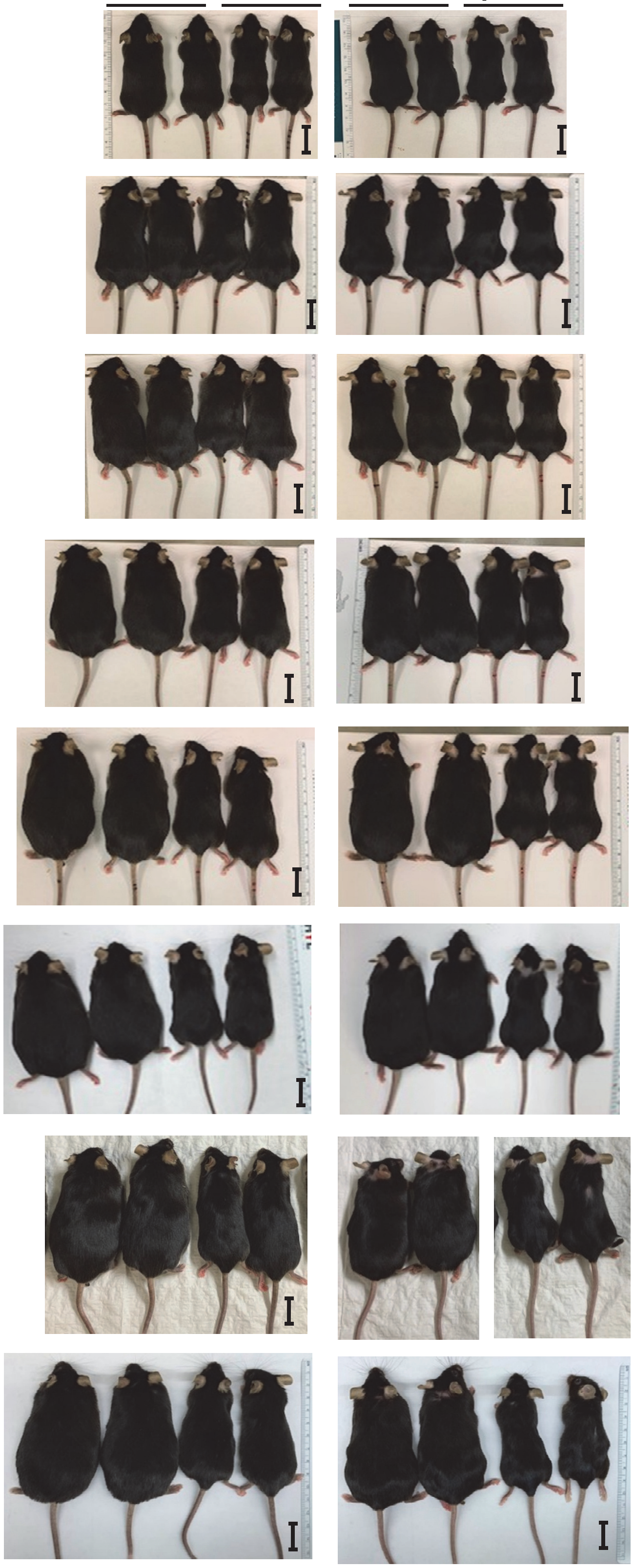

2 cm

Supplemental Figure 3. Photos of male and female *Son*$^{+/-}$ mice at different ages with gender-matched WT littermates at different ages. Next to the photos, the body weight and body length were indicated as average ± SD for each group. n=3-5 per group, p values from two-tailed, unpaired t-test were indicated for each group. Scale bar, 2 cm.

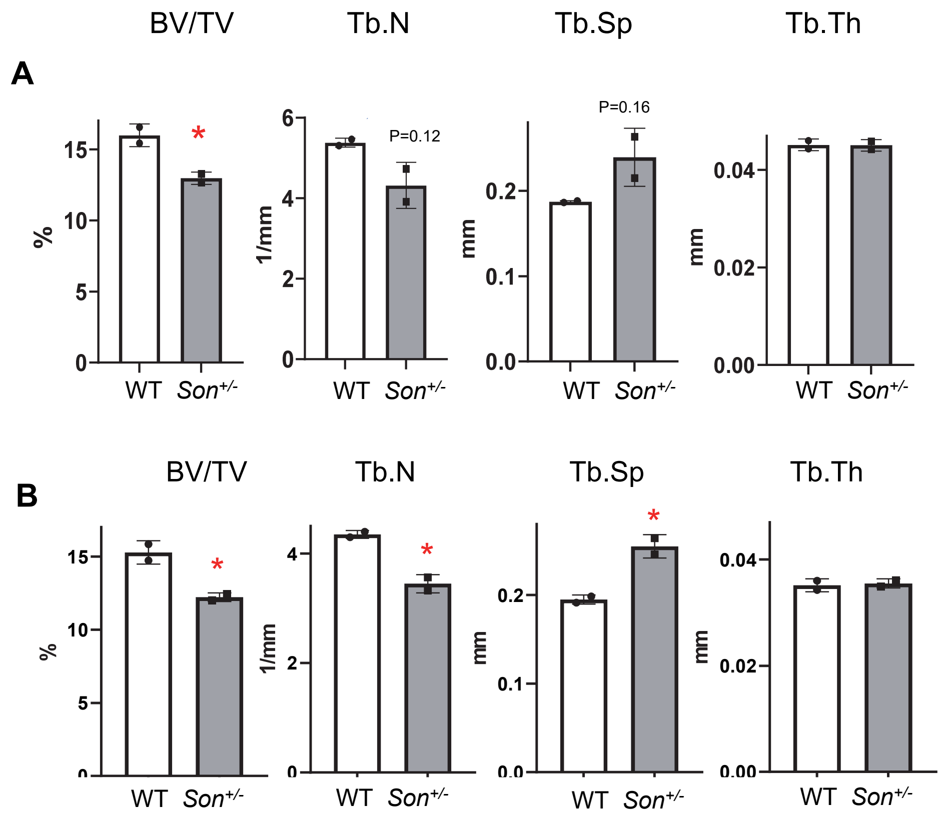

Supplemental Figure 4. Morphometric analyses of µCT data of femurs from 6-week-old female *Son*$^{+/-}$ and control mice using the TRI plate model of analysis. µCT Scans were reconstructed into 2D slices and all slices analyzed using the µCT Evaluation Program (v6.5-2, Scanco Medical) and 3D reconstruction performed using µCT Ray v4.2. Trabecular bone was evaluated in the distal femoral metaphysis (density threshold of 501 mg HA/cm3). For trabecular bone, the bone volume fraction (BV/TV), trabecular number (Tb.N), trabecular spacing (Tp.Sp), and trabecular thickness (Tb.Th) were determined using the DT analysis method (**A**) and TRI model analysis (**B**), according to the protocol https://www.scanco.ch/faq.html. n=2 per genotype, *$p < 0.05$.

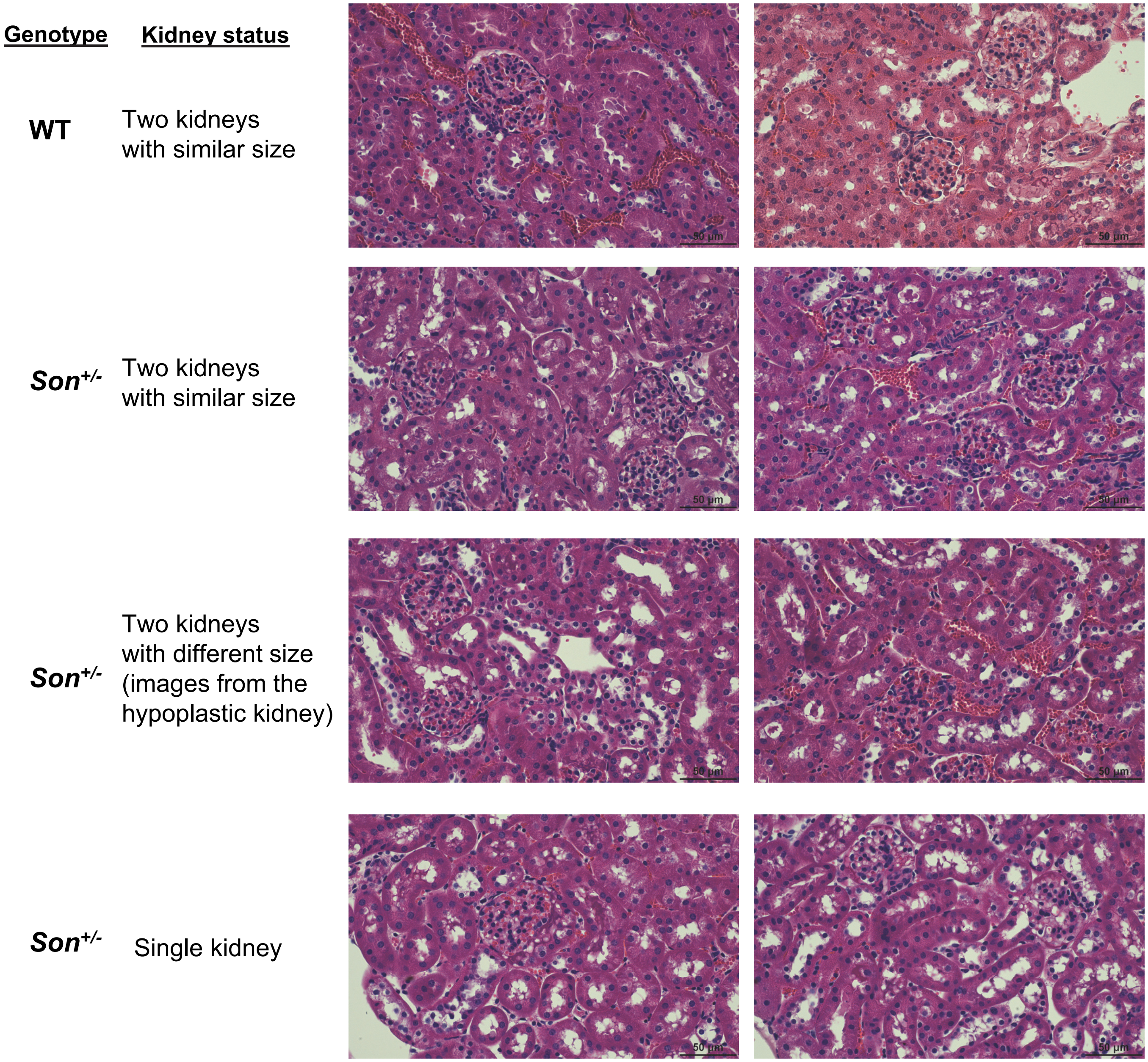

Supplemental Figure 5. H&E staining of the kidney sections from WT and *Son*$^{+/-}$ mice with indicated kidney status. Mild tubular regeneration and interstitial edema were observed in *Son*$^{+/-}$ kidneys, especially in the sample from *Son*$^{+/-}$ mice with a single kidney.

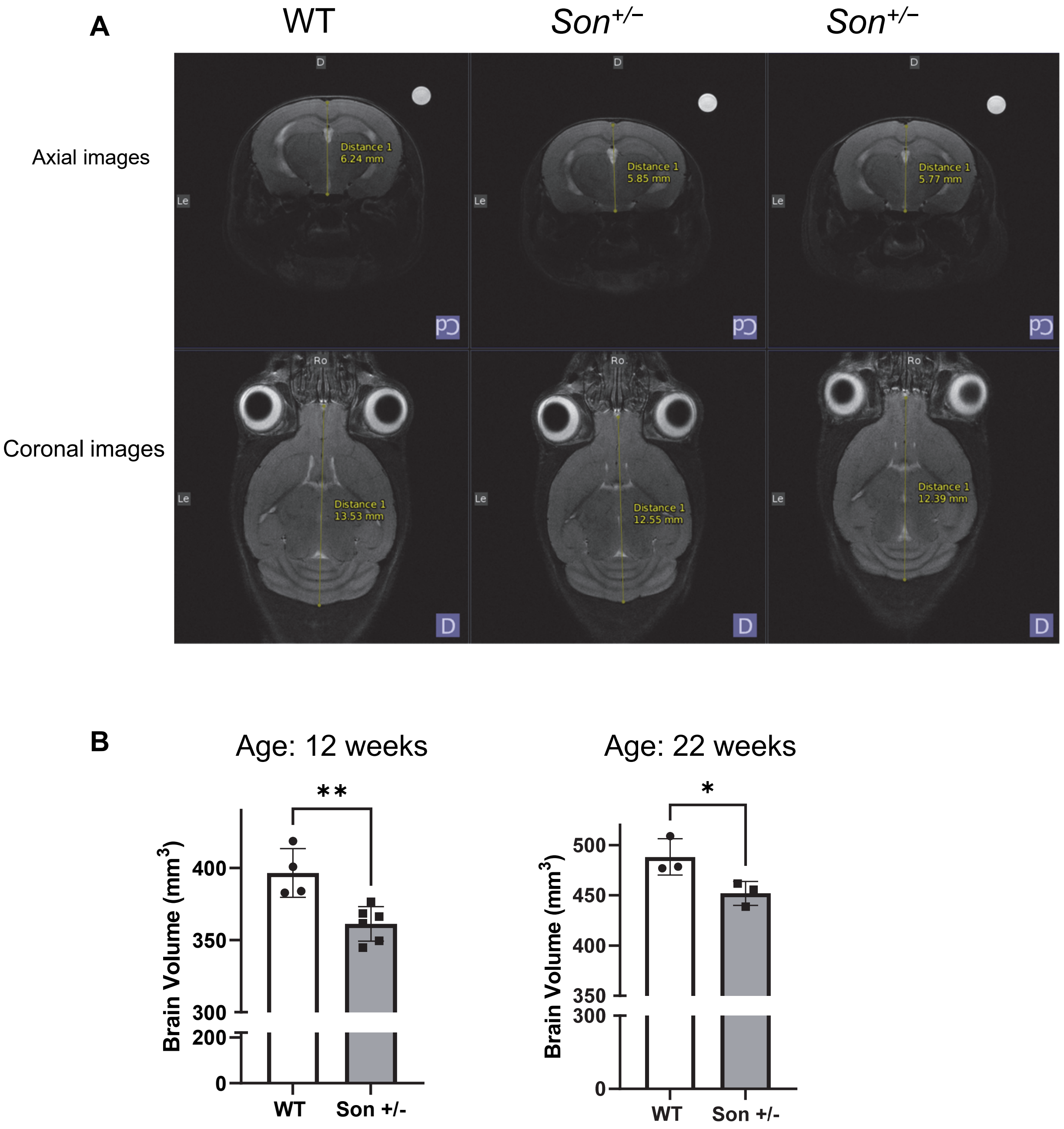

Supplemental Figure 6. Magnetic resonance imaging (MRI) of the brain indicates that *Son*$^{+/-}$ mice have smaller brain volumes compared to WT mice of matched age. (**A**) Representative T2-weighted brain MRI images of 10-week-old WT and *Son*$^{+/-}$ mice. MRI was performed using a preclinical 9.4T MRI scanner (Bruker BioSpin MRI GmbH, Ettlingen, Germany) with an 86 mm inner-diameter quadrature volume coil for excitation and a 2x2 brain surface coil array for signal reception. (**B**) Graphs indicating the brain volumes calculated from the MRI data. High-resolution T2-weighted images were acquired using a multi-slice rapid acquisition with relaxation enhancement (RARE) spin-echo pulse sequence with the following parameters: TR/TE = 2000 ms/40 ms; NEX = 8; RARE factor = 8; bandwidth = 66.7 kHz; FOV = (20 x 20) mm; matrix size = minimum of (256 x 256); slice thickness = 0.6-0.7 mm. Regions of interest (ROIs) were drawn around the entirety of the brain in each image slice using ImageJ Fiji. The area of each ROI was calculated using ImageJ Fiji and multiplied by the image slice thickness to yield an estimated volume measurement for the entire brain. Mean ± SD, * $p < 0.05$, ** $p < 0.01$.

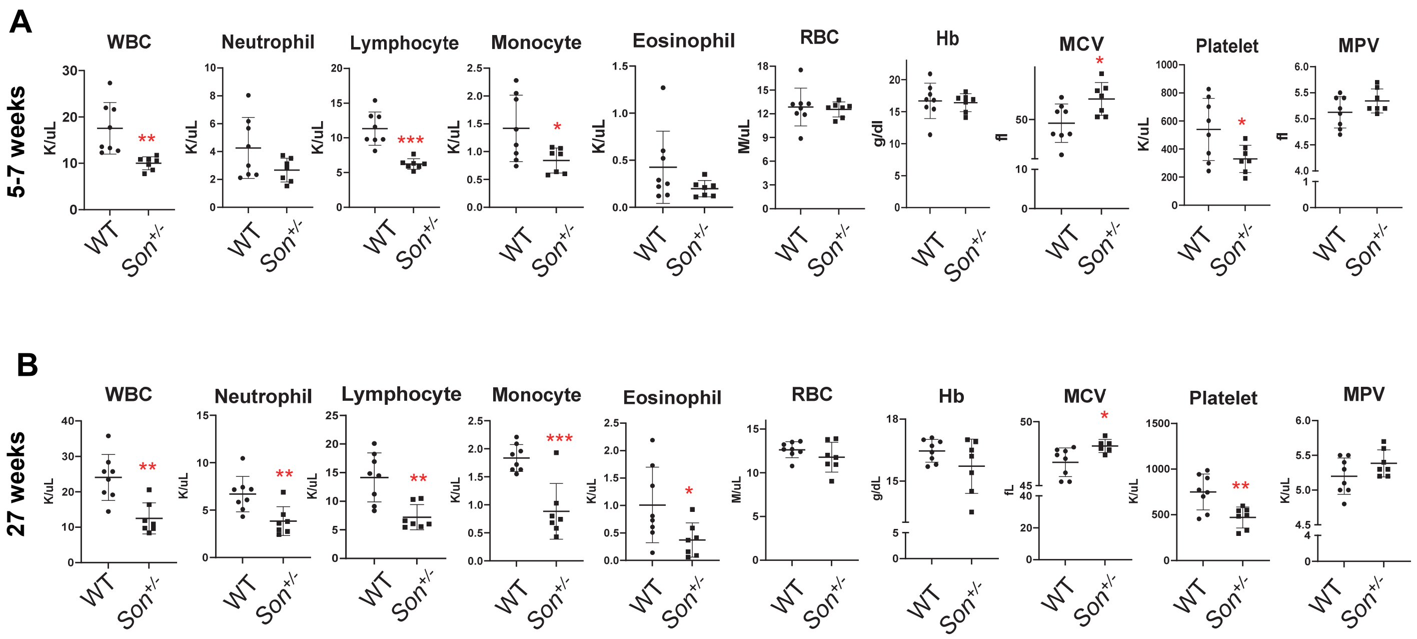

Supplemental Figure 7. Complete blood counts (CBC) from whole blood of the *Son*$^{+/-}$ mice showed the abnormalities which are similar to those observed in human ZTTK syndrome patients. CBC were performed for *Son*$^{+/-}$ mice and WT littermates at age 5-7 weeks (**A**) and 27 weeks. (**B**) Persistent abnormalities found in *Son*$^{+/-}$ mice include decrease of overall white blood cells (WBCs), neutrophils, monocytes, lymphocytes, and platelets. Red blood cell (RBC) counts are in normal range, but MCV is significantly increased in *Son*$^{+/-}$ mice, indicating impaired terminal differentiation of RBCs. Data are presented as mean ± SD, n=7-8.

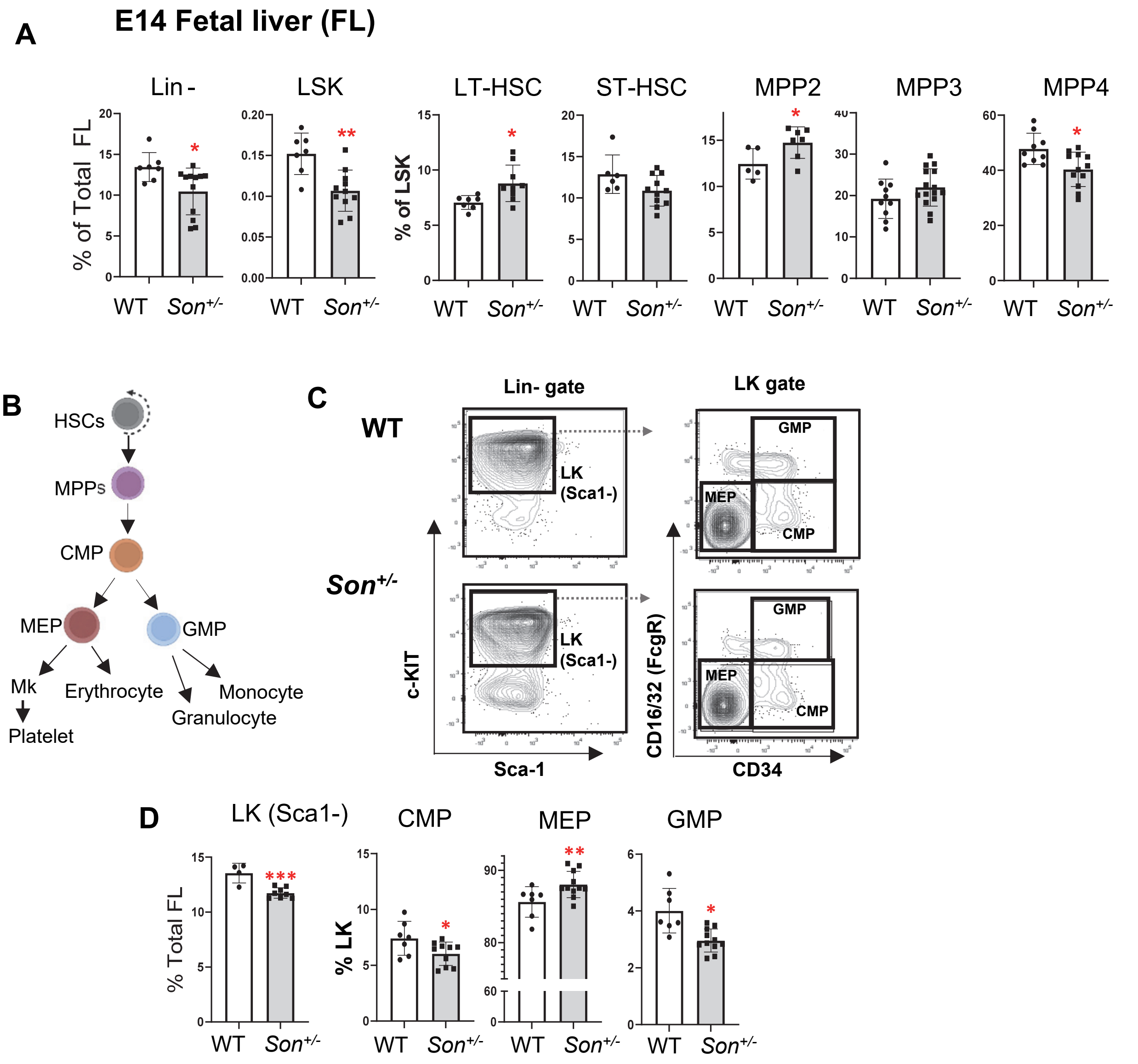

Supplemental Figure 8. Analyses of fetal liver hematopoiesis identified altered HSPC subpopulations and an imbalance of myeloid progenitor lineage fate in *Son*$^{+/-}$ embryo. (**A**) Frequency of the indicated populations within total fetal liver cells from E14 embryos (for Lin- and LSK) or within the LSK population (LT-HSC, ST-HSC, MPP2, MPP3 and MPP4). Data are expressed as mean ± SD, n=8-11, *p < 0.05, **p < 0.01, ***p < 0.001. (**B**) A schematic depicting the classical model of myeloid progenitor differentiation. (**C**) Flow cytometry contour plots showing the gating scheme for LK (Sca1-), CMP, GMP, and MEP. (**D**) Frequency of indicated populations within E14 fetal liver cells for LK (Sca1-) or within the LK population (for CMP, MEP, and GMP). Data are expressed as mean ± SD, n=5-11, *p < 0.05, **p < 0.01, ***p < 0.001.

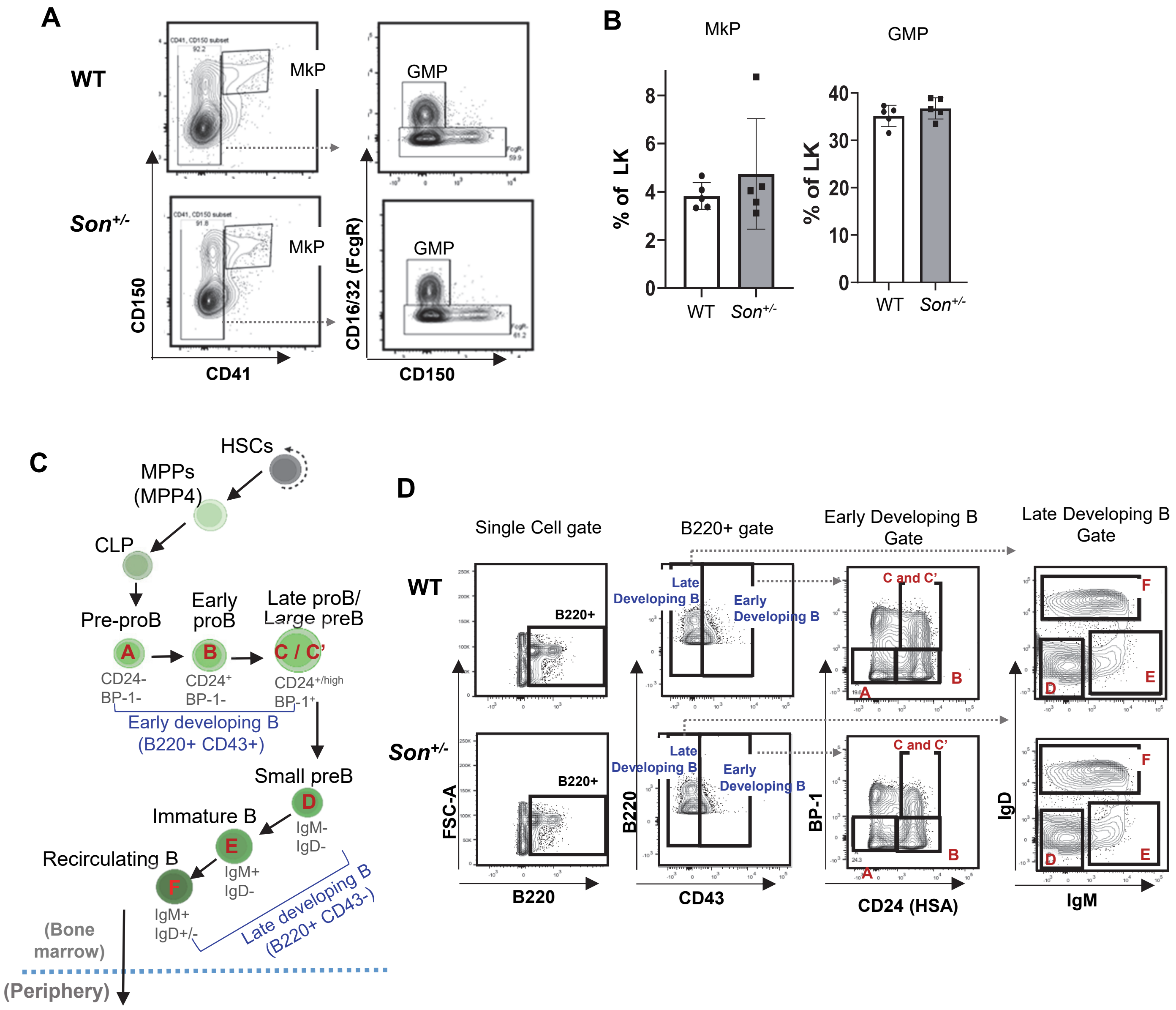

Supplemental Figure 9. Analyses of myeloid progenitors and B cell progenitor in the bone marrow. (**A**) Flow cytometry contour plots showing the gating scheme for MkP (megakaryocyte progenitors) and GMP (granulocyte/monocyte progenitors). (**B**) Frequency of MkP and GMP within bone marrow LK (Lin-, cKit+) cells, expressed as mean ± SD, n=5. (**C**) A schematic depicting the stages of B cell development in the bone marrow. Hardy fractions A – F (in red font, based on Hardy et al. 1991) are shown with surface markers. (**D**) Flow cytometry contour plots showing the gating scheme for Hardy fractions A – F, by determining the expression status of B220, CD43, CD24, BP-1, IgM, and IgD.

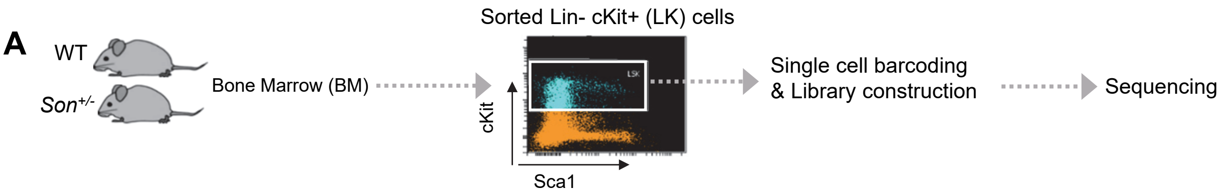

B

Sample	Number of analyzed cells	Number of annotated cells
Son$^{+/-}$-1	12,078	11,764
Son$^{+/-}$-2	19,341	19,468
WT-1	12,349	12,173
WT-2	12,412	12,182
Son$^{+/-}$ Total	31,521	31,232
WT Total	24,539	24,355

<u>Filteration criteria:</u>

- nUMI >= 500
- nGene >= 250
- log10GenesPerUMI > 0.80
- mitoRatio < 0.20

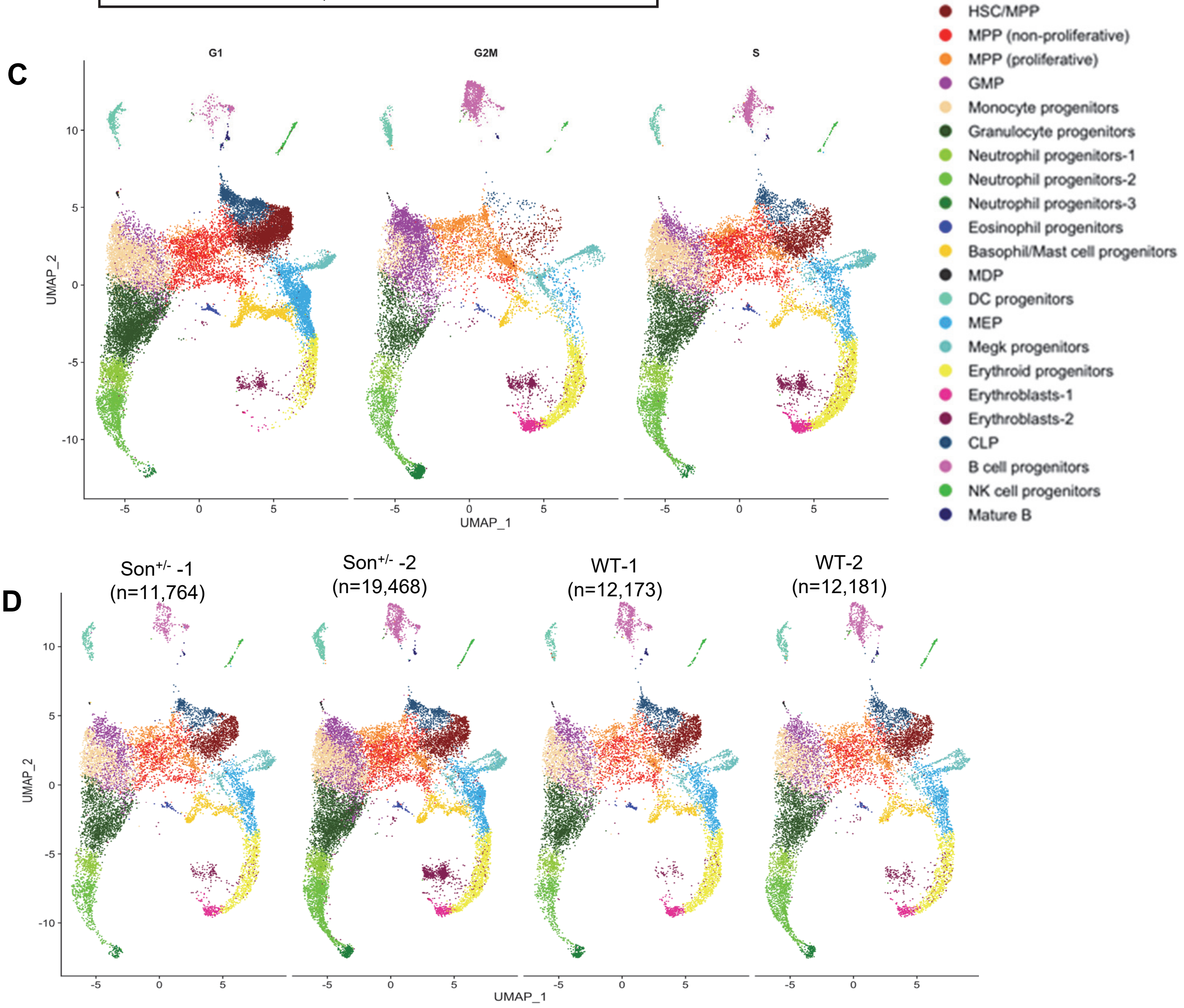

Supplemental Figure 10. Quality control, filtering criteria, and UMAP lots of single-cell RNA-sequencing (scRNAseq) data. (**A**) Schematic of the scRNAseq procedure. (**B**) Numbers of cells analyzed and annotated after filtration using the criteria listed. (**C**) Cell cycle status (G1, G2/M, and S phases) of the 22 clusters (color-coded) identified by Seurat. (**D**) UMAP plots showing the 22 clusters identified in the bone marrow LK cells from WT and *Son*$^{+/-}$ mice. The numbers of annotated/plotted cells per each sample are indicated.

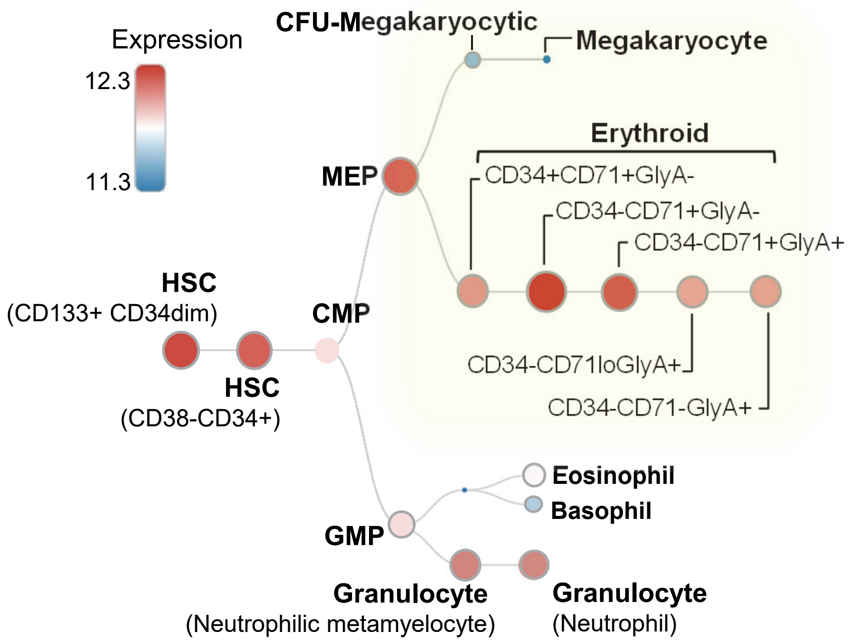

Supplemental Figure 11. SON expression in normal hematopoiesis. During normal hematopoiesis in humans, SON is highly expressed in differentiating erythroid cells, in sharp contrast to the much lower level detected in differentiating megakaryocytes. This suggests that a high level of SON expression is crucial for proper erythroid differentiation. The images are from the BloodSpot database (www.bloodspot.eu); Normal human hematopoiesis (DMAP) dataset for Probe 214988_s_at with selected hematopoietic populations.

Supplemental Table 1. Hematological abnormalities identified in human ZTTK syndrome patients*.

**Myeloid lineage phenotype**	**Megakaryocyte / Erythroid lineage**	• Large RBC (High MCV)<br>• High and Low RBC<br>• Polycythemia<br>• Thalassemia (Low Hemoglobin)<br>• Low Platelets<br>• Deep Vein Thrombosis (DVT) (Blood clots, Stroke)
	**Granulocyte / Monocyte lineage**	• High and Low WBC<br>• High and Low Neutrophils
**Lymphoid lineage phenotype**		• Low lymphocyte counts<br>• Immunoglobulin deficiency (IgG, IgA, and IgM)<br>• Poor response to vaccine<br>• Recurrent Infection (ex. respiratory infection, severe allergy, severe reactions to common illnesses, common variable immune deficiency (CVID))<br>• Intravenous Immunoglobulin (IVIG) monthly<br>• Autoimmune disorders (e.g. Autoimmune encephalitis)
**Others**		• Bone marrow failure<br>• High Vit. B12 in serum<br>• Low Vit. A and Vit. E

*Voluntary reports from patients and families to the ZTTK SON-Shine Foundation, https://zttksonshinefoundation.org/

Supplemental Table 2. Antibodies used for flow cytometry analysis and sorting.

Antibodies (anti-mouse)	Source	Identifier
Lineage antibody-**FITC** (clones: CD3 (17A2), CD11b (M1/70), CD45/B220 (RA3-6B2), Ly-6G (Gr-1) (RB6-8C5) and TER-119 (TER-119)	Tonbo Bioscience	Fisher catalog # 50-105-5256
c-Kit (CD117)-**APC** (clone 2B8)	BD Biosciences	BD catalog # BD553356
Sca-1-**PerCP-cy5.5** (clone D7)	Biolegend	Biolegend catalog # 108123
Flk2 (CD135)-**PE** (clone A2F10)	Biolegend	Biolegend catalog # 135305
CD150-**PE-cy7** (clone mShad150)	Invitrogen	Fisher catalog # 50-245-735
CD48-**APC-Cy7** (clone HM48-1)	BD Biosciences	BD catalog # BD561242
CD16/32 (FcRII)-**APC-cy7** (clone 93)	Biolegend	Biolegend catalog # 101327
CD41-**BV711** (clone MWReg30)	BD Biosciences	BD catalog # BDB740712
CD71-**PE** (clone C2)	BD Biosciences	BD catalog # BDB553267
CD105 (Endoglin)-**BV605** (clone MJ7/18)	BD Biosciences	BD catalog # BDB740425
Ter-119-**PE-cy5** (clone TER-119)	Invitrogen	Fisher catalog # 15592181
CD45R/B220-**Pacific Blue** (clone RA3-6B2)	BD Biosciences	BD catalog # BDB558108
BP-1/Ly-51-Biotin (clone 6C3)	Biolegend	Fisher catalog # 108303
CD43-**FITC** (clone S7)	BD Biosciences	BD catalog # BDB553270
CD24-**PE** (clone M1/69)	BD Biosciences	BD catalog # BDB553262
CD23-**PE-Cy7** (clone B3B4)	eBioscience	Fisher catalog # 50-154-79
IgD-**PerCP-eFluor 710** (clone 11-26c (11-26))	eBioscience	Fisher catalog # 50-112-8623
IgM-**APC** (clone II/41)	eBioscience	Fisher catalog # 50-152-13
CD21-**FITC** (clone 7G6)	BD Pharminogen	Fisher catalog # 561769
CD93-**PE** (clone AA4.1)	Invitrogen	Fisher catalog # 12-5892-81
CD34-**PE Dazzle 594** (clone MEC14.7)	Biolegend	Fisher catalog # 50-207-0869

Supplemental Methods

Genotyping

Tail or ear snips were incubated in 113 µL 50 mM NaOH at 95°C for 30 minutes, followed by adding 32 µL of 1M Tris buffer (pH 8.8) and centrifugation for 10 minutes. The supernatant was used for the following PCR reaction: 95°C for 5 minutes initiation, 95°C for 30 seconds, 58.5°C for 30 seconds, 72°C 1 kb/min for 35 cycles for elongation, and 72°C for 10 minutes for termination. Primer sequences to detect the presence of the LoxP sequence are the following: 3' LoxP site (Forward: AGAACATGGCCACCCATTTCTTCCA, Reverse: ATCCCTCTCTAGGAGTTGCTGGTGT) and 5' LoxP site (Forward: TGCCACTTGTTCTGTGTAAAATTCTTGA, Reverse: GCCACACTGTGCACTGATCACAAAC). To detect the deletion of the LoxP-floxed sequence, the forward primer for 5' LoxP and the reverse primer for 3' LoxP listed above were used.

Analysis of single-cell RNA-sequencing data

Cellranger-7.1.0 (10X Genomics) pipeline was used to align the raw sequencing data to the mm10 mouse reference genome and count the expressed transcripts. Seurat (v4.3.0) was used to analyze and visualize the processed files. For quality control and filtration, cells were filtered using four criteria; (a) the gene number per cell to be more than 250, (b) the UMI counts of more than 500 per cell, (c) log10 genes per UMI of more than 0.80, and (d) mitochondrial gene expression per cell less than 20% of total gene expression. Normalization and variance stabilization of filtered row counts were done using the SCtransform method to account for the variation due to sequencing depth.

To ensure that the cell types of wild-type align with the same cell types of knockout, samples were integrated using shared highly variable genes from each condition. The top 3000 highly variable features were selected using *SelectIntegrationFeatures* and *PrepSCTIntegration* functions. They have been used to perform canonical correlation analysis (CCA) to correct the

batch effect and to find anchors between two datasets using the mutual nearest neighbors (MNNs) algorithm using *FindIntegrationAnchors*. The final integration step was done by the *IntegrateData* function.

For clustering analysis and annotation, a graph-based clustering method using a K-nearest neighbor algorithm (*FindNeighbors* function) was applied based on the top 40 PCA, then performing the clustering step using the *FindClusters* function to group cells together with a resolution of 0.6. Cell clusters were visualized using UMAP by first applying the *RunPCA* function, then *RunUMAP* with dims parameter 1:40. Clusters annotation was done manually based on the conserved marker genes identified using the *FindConservedMarkers* function. Then, these genes were used in the mouse hematopoietic marker gene sets database (CellKb Immune v2.3) to label the clusters.
