# Supplementary material for: A mouse model of Zhu-Tokita-Takenouchi-Kim syndrome reveals indispensable SON functions in organ development and hematopoiesis
Source: JCI Insight. 2024 Jan 30;9(5):e175053. doi: 10.1172/jci.insight.175053 (PMC10972584; doi:10.1172/jci.insight.175053)

Figure 1I

Lung_Son

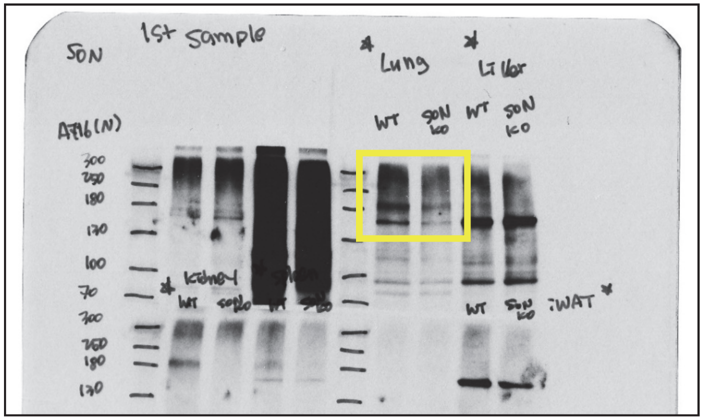

Lung_β-tubulin

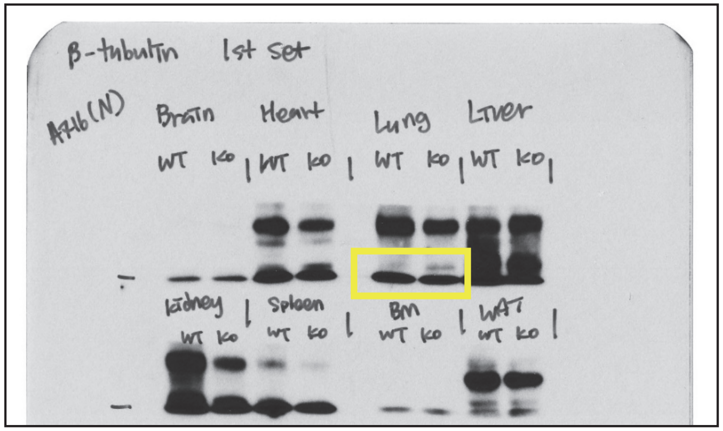

Figure 1I

Kidney_Son

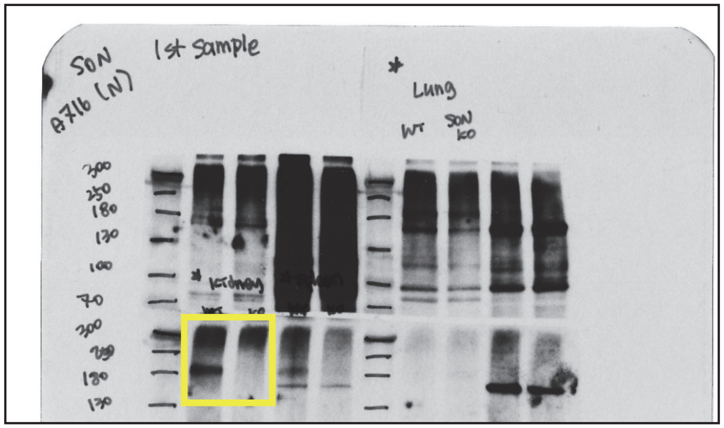

Kidney_β-tubulin

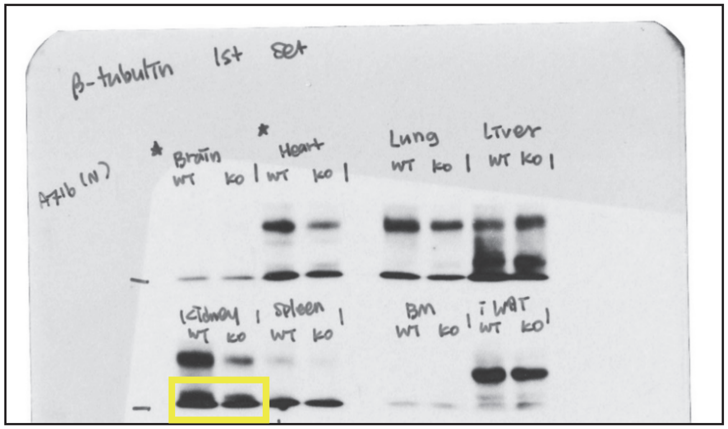

Figure 1I

Spleen_Son

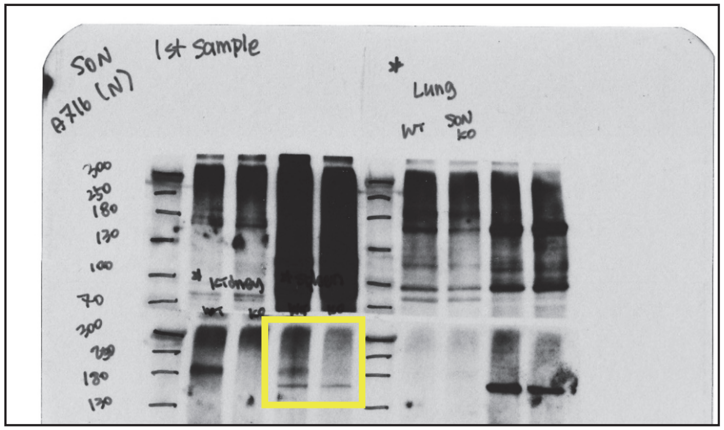

Spleen_β-tubulin

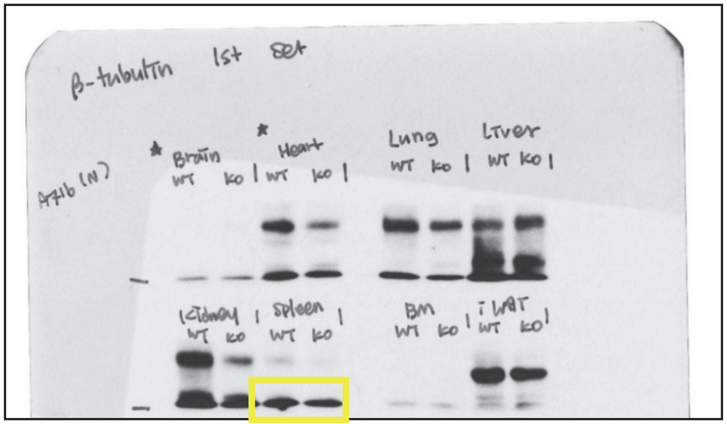

Figure 1I

Liver_Son

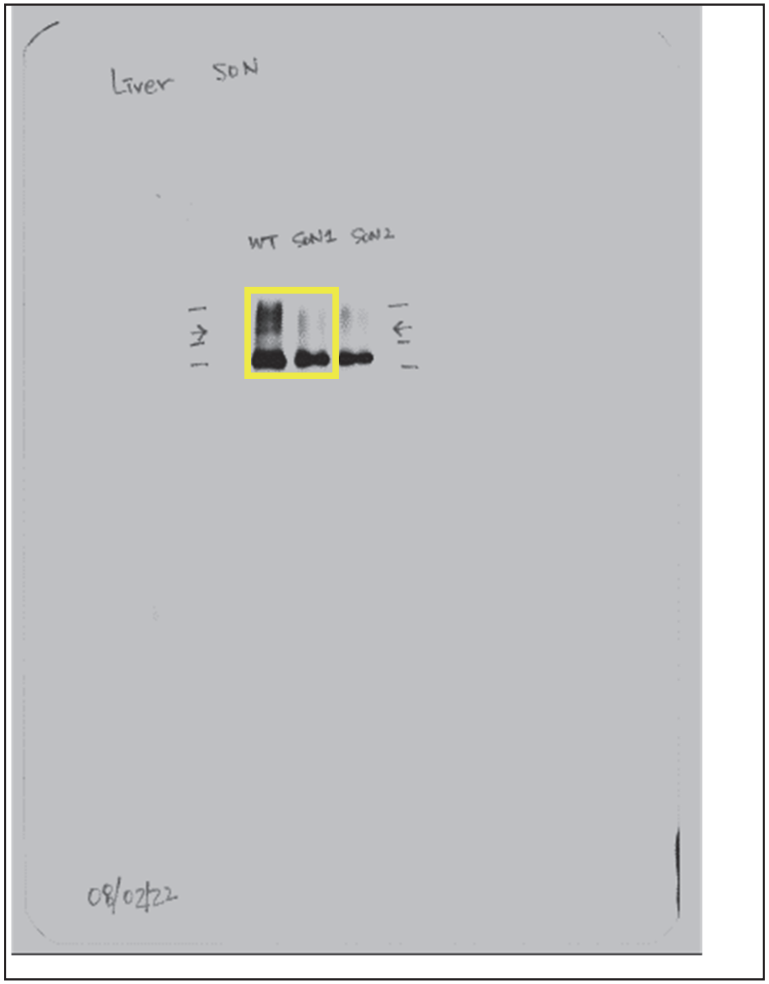

Liver_β-actin

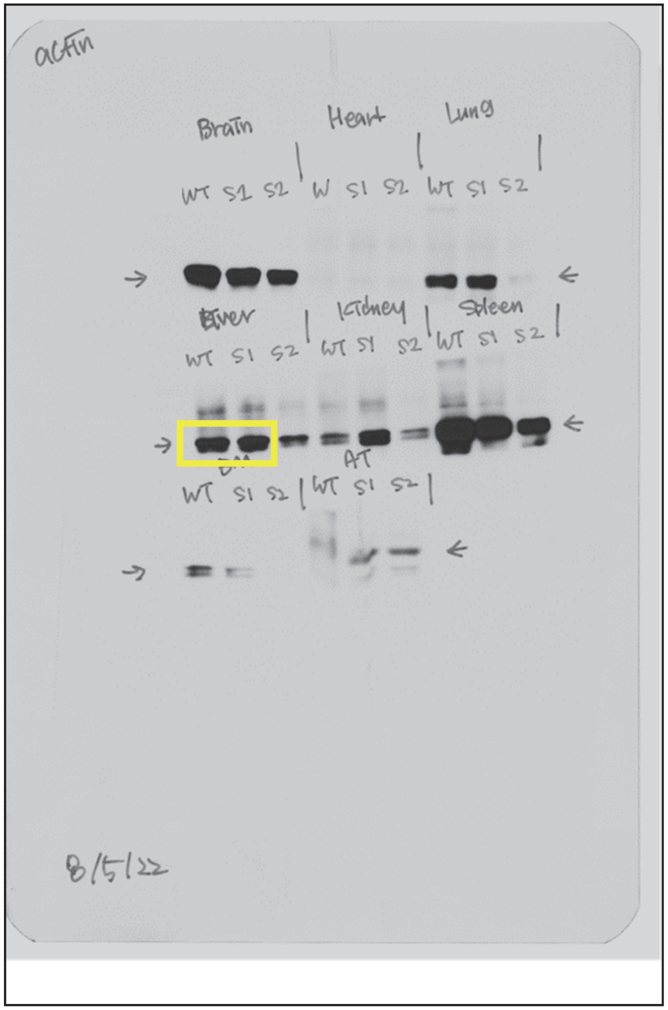

Supplemental Figure 2D

Brain_Son

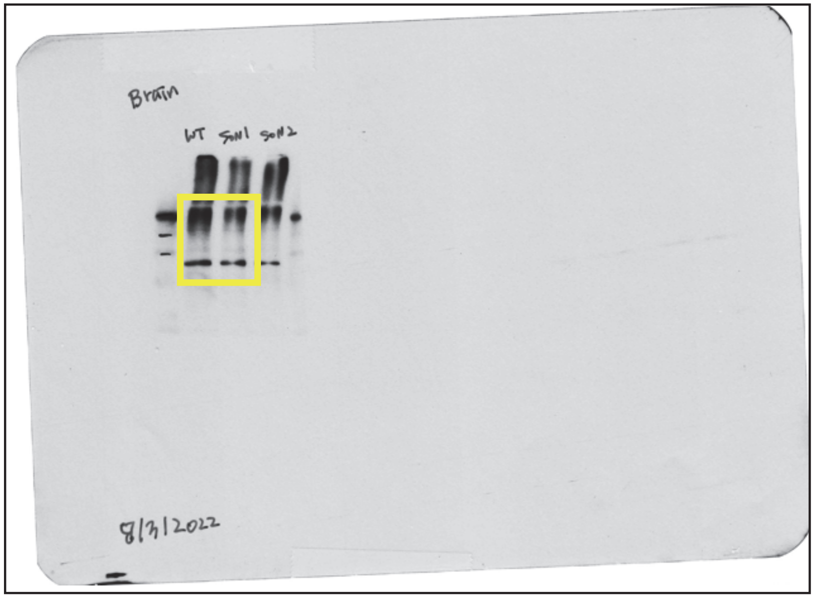

Brain_β-actin

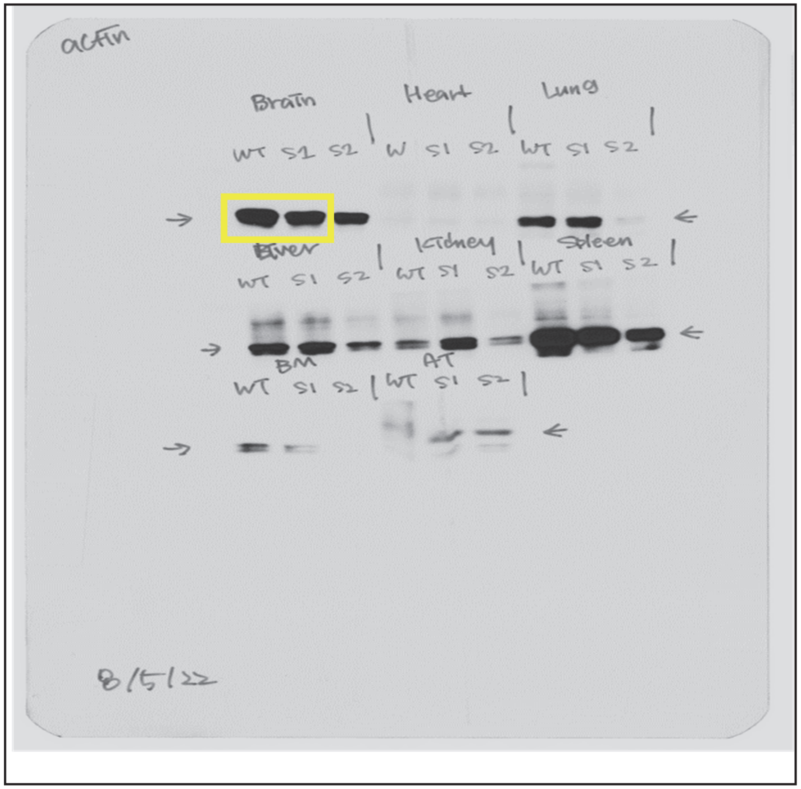

Supplemental Figure 2D

Heart_Son

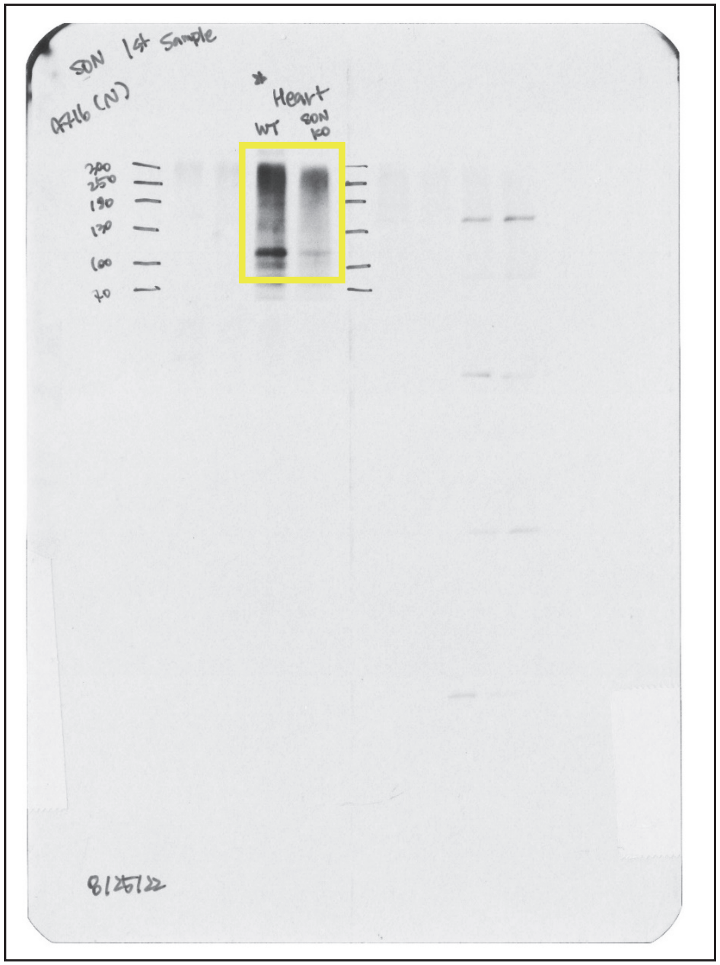

Heart_β-tubulin

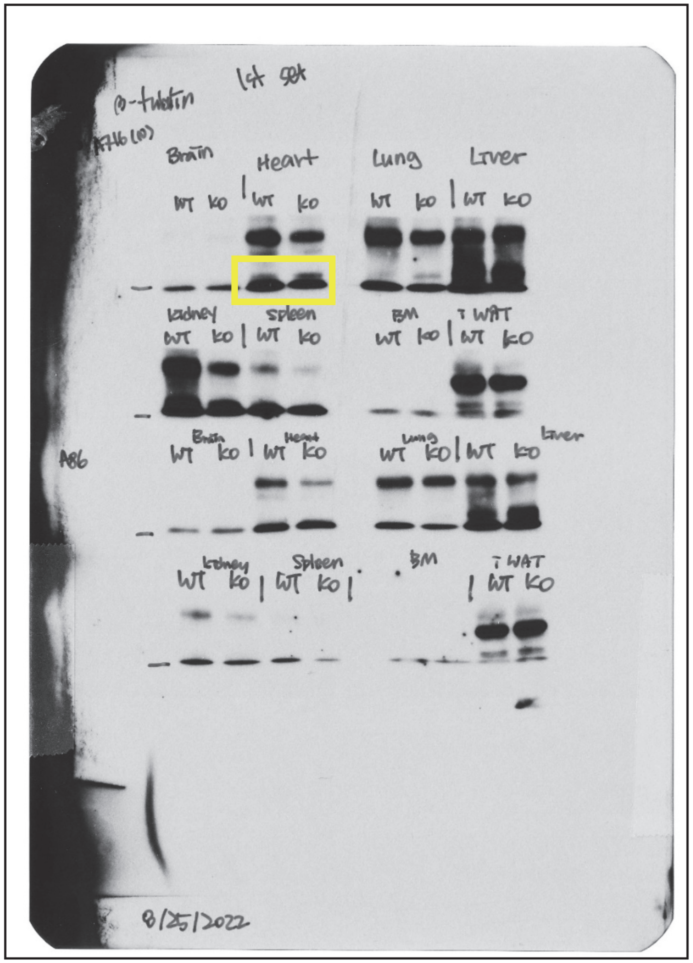

Supplemental Figure 2D

Bone marrow_Son

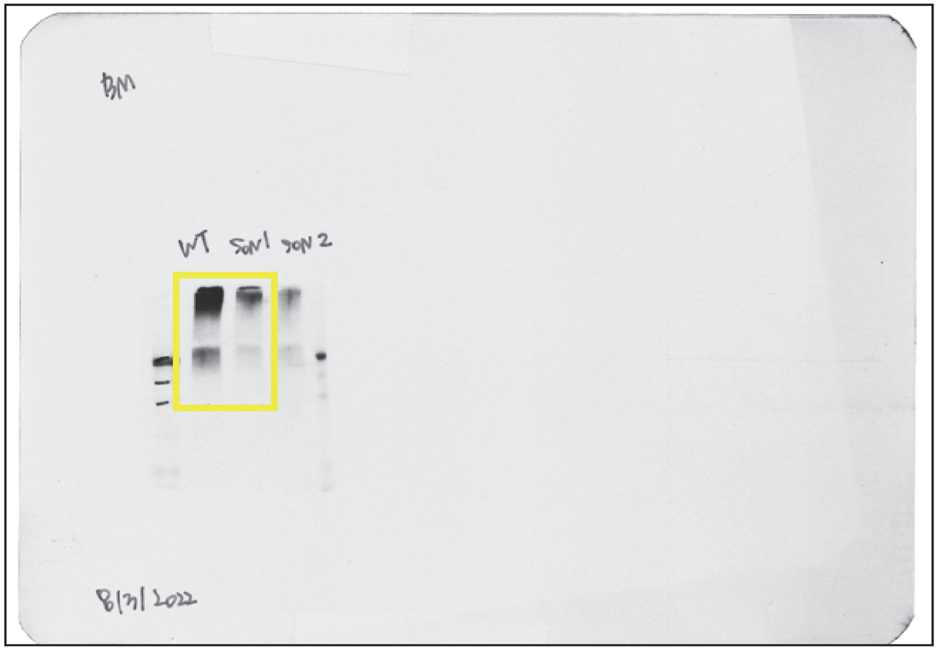

Bone marrow_β-actin

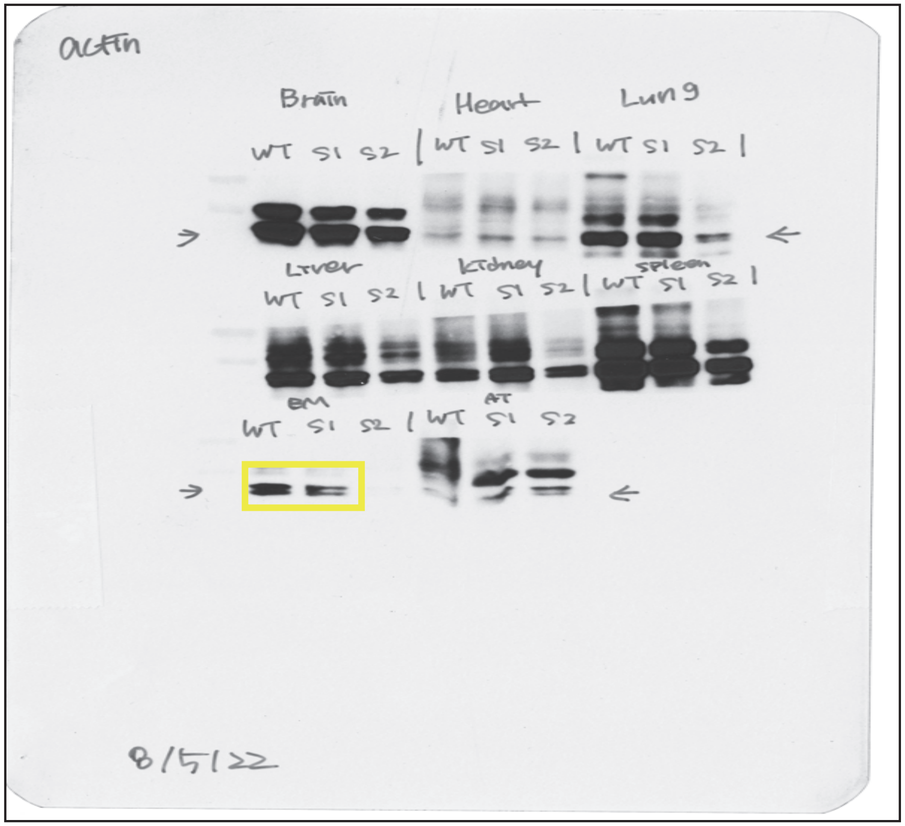

Supplement: Unedited blot and gel images [file jciinsight-9-175053-s067.pdf]
